# H_2_O_2_ Synthesis with Molecular Electrocatalysts Enables Substantial Current Densities in a Flow Cell Using Gas Diffusion Electrodes

**DOI:** 10.1021/acselectrochem.5c00179

**Published:** 2025-10-08

**Authors:** Phebe H. van Langevelde, Nathalie E. G. Ligthart, Pim G. J. van Duren, David A. Vermaas, Dennis G. H. Hetterscheid

**Affiliations:** † Leiden Institute of Chemistry, Leiden University, 2300 RA Leiden, The Netherlands; ‡ Department of Chemical Engineering, Delft University of Technology, 2629 HZ Delft, The Netherlands

**Keywords:** Hydrogen Peroxide, Gas Diffusion Electrodes, Rotating Ring Disk Electrodes, Homogeneous Catalysis, Copper Complexes

## Abstract

The development of catalysts for the two-electron oxygen reduction reaction (ORR) toward H_2_O_2_ generation has gained substantial interest, as well as studying their activity in advanced electrochemical cell configurations. However, molecular ORR catalysts are seldom studied outside of the idealized environment of a rotating disk electrode (RDE) setup. Consequently, the catalytic currents reported for all molecular catalysts are limited by the mass transport of oxygen, and their properties in industrially relevant reactor configurations have remained unexplored. To assess their full potential, we report herein the application of a molecular, copper-based ORR catalyst, Cu­(tmpa) (tmpa = tris­(2-pyridylmethyl)­amine), in a cell configuration with gas diffusion electrodes (GDEs) to enhance mass transfer and thereby catalytic currents. We have identified the factors that control the catalytic current, such as buffer and catalyst concentration and GDE composition, using a readily assembled GDE cell, and we demonstrate that Cu­(tmpa) can generate H_2_O_2_ over multiple hours in a GDE flow cell. Under these conditions, a Faradaic efficiency of 50% can be maintained and H_2_O_2_ can be generated at a rate of 0.11 mmol cm^–2^ h^–1^ at a current density of −20 mA/cm^2^. The current density is increased by almost 10 times in a GDE configuration compared to a conventional RDE setup, and the selectivity trends of the GDE system differ from the results obtained in an RDE setup, as a higher current density generates a higher selectivity toward H_2_O_2_ in a GDE setup. Overall, this work shows that the full potential of molecular catalysts can be assessed upon their implementation in a GDE setup and that their well-defined active sites can contribute to the emerging field of H_2_O_2_-generating catalysts.

## Graphical abstract

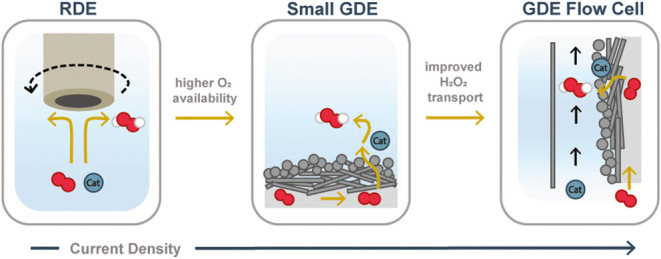

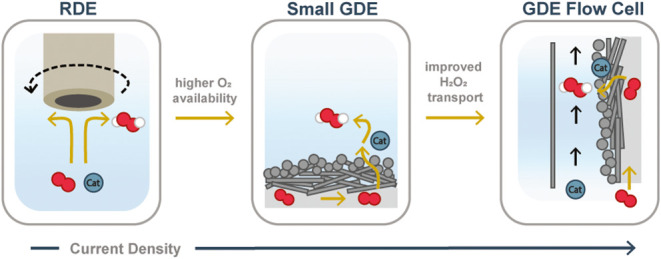

## Introduction

Hydrogen peroxide is an environmentally benign oxidizing agent because of its high percentage of active oxygen and because H_2_O_2_ only generates water as a byproduct in oxidation reactions.^1^ However, the anthraquinone process is responsible for 95% of the global hydrogen peroxide production at the moment, but this process is not considered sustainable.^2^ The anthraquinone method is a batch process that contains various energy-intensive purification steps. Furthermore, its large-scale and centralized character require that H_2_O_2_ solutions are concentrated up to 70 wt % for transport, which not only raises safety concerns but also is impractical as many applications only require the use of H_2_O_2_ in a strongly diluted form.^3^ Considering these disadvantages, the decentralized production of hydrogen peroxide is a viable alternative. A promising strategy for the on-site synthesis of H_2_O_2_ entails the two-electron reduction of oxygen.^4^ This approach facilitates the generation of H_2_O_2_ under ambient conditions in quantities tailored to the desired application. Moreover, the electrochemical oxygen reduction reaction (ORR) can be coupled to renewable energy sources, like solar or wind energy, to make the production process fully renewable.^5^

The reduction of oxygen is a complex, multistep reaction that can proceed through various pathways. A two-electron reduction generates H_2_O_2_ (eq 1), but oxygen can also be reduced to H_2_O in a thermodynamically more favorable 4-electron process (eq 2). In addition, many catalysts are capable of reducing hydrogen peroxide further in an additional two-electron process known as the hydrogen peroxide reduction reaction (HPRR) (eq 3).

1 $$O_{2}+2e^{-}+2H^{+}\to H_{2}O_{2}\;\left(2‐\mathrm{electron}\;\mathrm{ORR},0.70\;V\;\mathrm{vs}\;\mathrm{NHE}\right)$$

2 $$O_{2}+4e^{-}+4H^{+}\to 2H_{2}O\;\left(4‐\mathrm{electron}\;\mathrm{ORR},1.23\;V\;\mathrm{vs}\;\mathrm{NHE}\right)$$

3 $$H_{2}O_{2}+2e^{-}+2H^{+}\to 2H_{2}O\;\left(\mathrm{HPRR},1.76\;V\;\mathrm{vs}\;\mathrm{NHE}\right)$$

In recent years, there has been a growing interest to develop catalysts for the two-electron ORR for H_2_O_2_ generation.^4^ A wide variety of heterogeneous catalysts has been reported for this purpose, ranging from noble metals and their alloys, often based on Pt or Pd,^6–11^ to carbonaceous materials,^12–14^ potentially doped with heteroatoms,^15–17^ or metals to create single-atom catalysts (SACs).^18–24^ However, elucidating structure-activity relationships from these catalysts poses challenges due to the often ill-defined surface of heterogeneous catalysts,^25^ and clear identification of the active sites of H_2_O_2_-generating catalysts is stated as a challenge in the field.^12,26–28^

In contrast, molecular catalysts typically offer a well-defined active site at which ligands interact with a metal center, and the secondary coordination sphere is well understood. Moreover, the ligand structure of molecular systems can be rationally tuned to study the role of the ligand in the reactivity of the catalyst. With regard to the ORR, the activation of oxygen at various metal sites has been very well documented,^29–33^ and many catalysts based on first-row transition metals for ORR in aqueous solution have been reported.^34,35^ Examples of these catalysts include metalloporphyrins based on the active site of cytochrome c oxidase^36–40^ and pyridylalkylamine complexes inspired by the active site of multicopper oxidases.^41–52^ Effort has been made to improve the activity and stability of molecular catalysts in electrochemical configurations. However, few studies have reported molecular, homogeneous ORR catalysts that are able to generate H_2_O_2_ over extended periods of time.^53–57^ We observed excellent activity and good stability of a copper-based molecular catalyst generating H_2_O_2_ with a Faradaic efficiency (FE_H2O2_) over 50% during 8 h in previous work (Figure 1).^58^

**Figure 1:**
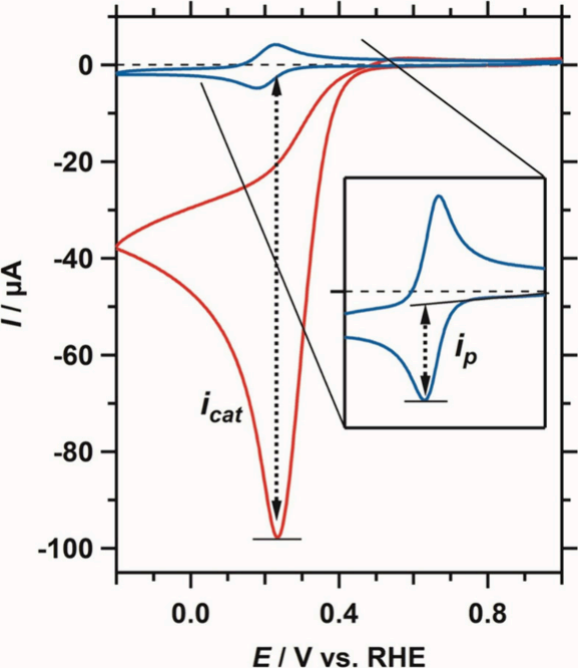
Cyclic voltammetry of 0.3 mM of dissolved Cu­(tmpa) in the absence (blue) and presence (red) of O_2_ (1 atm) in a 0.1 M phosphate buffer under stationary conditions. Adapted with permission from ref 41.

In most studies, these molecular catalysts are evaluated in simple three-electrode setups instead of realistic and scalable systems, which is in contrast with the heterogeneous catalytic systems currently explored for practical H_2_O_2_ generation that require much higher current densities. Homogeneous flow reactors suffer from mass transport limitations of dissolved oxygen, illustrated by limiting current densities ranging from <1 mA/cm^2^ at a flat-plate electrode to 10–20 mA/cm^2^ at a porous flow-through electrode.^59^ To further enhance the mass transport to industrial standards, we need to study advanced electrochemical cell configurations such as gas diffusion electrodes (GDEs) and membrane electrode assemblies,^60^ similar to those of a fuel cell or electrolyzer.^61,62^

Gas diffusion electrode (GDE) configurations circumvent the low solubility and slow transport of oxygen in electrolyte, because it allows diffusion of O_2_ through the electrode toward the catalyst from the gaseous side.^63^ Because the O_2_ concentration is 40× higher in the gas phase compared to water (at 1 bar) and its diffusivity is four orders of magnitude higher in the gas phase, the GDE allows alleviation of mass transport limitations. As to fully assess the potential of molecular catalysts, molecular ORR catalysts should therefore be studied using GDEs. Over the last ten years, around 20 studies have reported molecular catalysts in combination with GDEs, mainly focussing on CO_2_ reduction.^64^ Especially, porphyrin and phthalocyanine structures based on Co, Ni, or Fe have been shown to be excellent catalysts for CO_2_ reduction that can reach industrially relevant current densities of up to −400 mA/cm^2^ and can remain active over multiple days.^65–69^ However, to our knowledge, only one ORR study involving GDEs has been reported.^55^ In this work, cobalt tetraphenylporphyrin was used to generate H_2_O_2_ in a GDE setup, but the FE of this system was not reported, and the stability of the catalyst was not investigated.

Herein, we have taken the next step in H_2_O_2_ generation utilizing molecular catalysts by studying their activity, selectivity, and stability in a GDE setup. To do so, we have selected the mononuclear, copper-based catalyst Cu­(tmpa) (tmpa = tris­(2-pyridylmethyl)­amine). This catalyst is among the fastest molecular ORR catalysts reported to date with a maximum turnover frequency for the ORR of over 2 million per second;^41^ moreover, it can generate H_2_O_2_ electrochemically over multiple hours with a good FE_H2O2_ in a rotating disk electrode (RDE) setup^58^ and in a flow cell system.^59^ It should be pointed out that Cu­(tmpa) is able to further reduce H_2_O_2_ in the HPRR,^70^ but the observed rate constant of the two-electron ORR is more than 10 times higher than the rate constant of the HPRR. Therefore, it is anticipated that Cu­(tmpa) will catalyze the ORR with a high selectivity to H_2_O_2_, as long as the local concentration of O_2_ will remain high compared to the local H_2_O_2_ concentration.^58^

In this work, a GDE cell was used to identify which factors control the catalytic current such as buffer strength, GDE material, and catalyst concentration. Thereafter, a GDE flow cell was utilized to show that Cu­(tmpa) is able to generate H_2_O_2_ over multiple hours, establish a good FE of almost 60%, and reach current densities of up to −40 mA/cm^2^. This represents the first study in which such substantial current densities are obtained by a molecular catalyst that is dissolved in the liquid phase. Additionally, Cu­(tmpa) was heterogenized onto the GDE surface to improve the catalytic performance. Overall, the current densities obtained with such a GDE configuration exceeded the current densities obtained by a RDE setup by more than an order of magnitude and H_2_O_2_ could be generated over several hours at a rate of 0.11 mmol cm^–2^ h^–1^. Such rates are unprecedented for molecular oxygen reduction catalysts, producing hydrogen peroxide.

## Methods

Previously, the activity and selectivity of Cu­(tmpa) for the electrochemical generation of H_2_O_2_ were extensively studied in a rotating disk electrode (RDE) configuration.^58^ In an RDE setup, the flow of electrolyte and O_2_ are precisely controlled, which allows us to study the effect of mass transport on the ORR selectivity. It was established that a high selectivity for H_2_O_2_ up to 80% can be obtained in the presence of micromolar catalyst concentrations in this setup as long as the local O_2_ concentration remains high. However, the current density in an RDE configuration is limited by the mass transport of O_2_ toward the electrode. This mass transport, and therefore the current density, can be substantially improved in a GDE setup. In this work, the catalytic activity and selectivity were studied in two different GDE setups and compared to the RDE system. The reason these three setups were chosen is as follows:

1. RDE setup: The generation of H_2_O_2_ by Cu­(tmpa) in an RDE setup was studied in previous work^58^ and is therefore a suitable starting point to compare to gas diffusion electrode setups.
2. Small GDE setup: A small GDE cell, which was developed in the Arenz group^71–74^ and is now commercially available, was used to optimize the conditions for H_2_O_2_ generation. The setup is easily assembled, requires only small amounts of electrolyte, and has an electrode surface of only 0.071 cm^2^. In this study, this cell was mainly used to rapidly explore the reaction conditions to achieve maximum catalytic activity. This configuration is not suitable for the generation of H_2_O_2_ in bulk electrolysis, because the electrolyte does not flow.
3. GDE flow cell setup: A GDE in a flow cell configuration was utilized to generate H_2_O_2_ during electrolysis for multiple hours and determine the FE_H2O2_. The flow of the electrolyte enables the transport of H_2_O_2_ away from the electrode. The electrode surface of 3.8 cm^2^ is much larger than of the other two setups and allows more H_2_O_2_ to be generated.

A schematic overview of these setups is presented in Figure 2, and the exact details of the cell configurations can be found in Supporting Information Section 1. In both GDE setups, a GDE is termed the commercially available electrode material consisting of a gas diffusion layer (GDL) and in most cases a microporous layer (MPL). Contrasting with the field of heterogeneous catalysis, the GDEs discussed herein do not have a catalyst layer (CL), except in the case where the catalyst is heterogenized on the electrode surface. In all other experiments, including the RDE measurements, Cu­(tmpa) is dissolved in the electrolyte.

**Figure 2:**
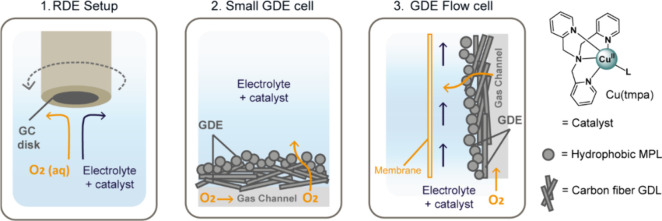
Overview of the electrochemical setups used in this work to generate H_2_O_2_ via the electrocatalytic ORR with Cu­(tmpa) as a homogeneous catalyst. From left to right: A rotating disk electrode (RDE) setup used a GC disk working electrode (**1**), a small gas diffusion electrode (GDE) cell (**2**), and a GDE flow cell in which the anode and cathode sides are separated by a membrane (**3**). The GDE used in the majority of experiments consists of a carbon fiber gas diffusion layer (GDL) with a hydrophobic microporous layer (MPL). On the right, a schematic representation of the catalyst used in this work, Cu­(tmpa), is presented in which L represents a solvent molecule coordinating to the Cu­(II) center.

## Results and Discussion

### Screening of Electrochemical Conditions

To shed light on the ORR activity of Cu­(tmpa) in a GDE setup, we first investigated the effect of the electrochemical conditions so as to optimize the electrochemical synthesis of H_2_O_2_. The activity of Cu­(tmpa) was investigated in the readily assembled small GDE setup by changing the GDE material, catalyst concentration, buffer concentration, and electrolyte composition (Figure 3). Impedance measurements were carried out, and full *iR* compensation was applied to all measured CV data unless stated otherwise (Supporting Information Section 2).

**Figure 3:**
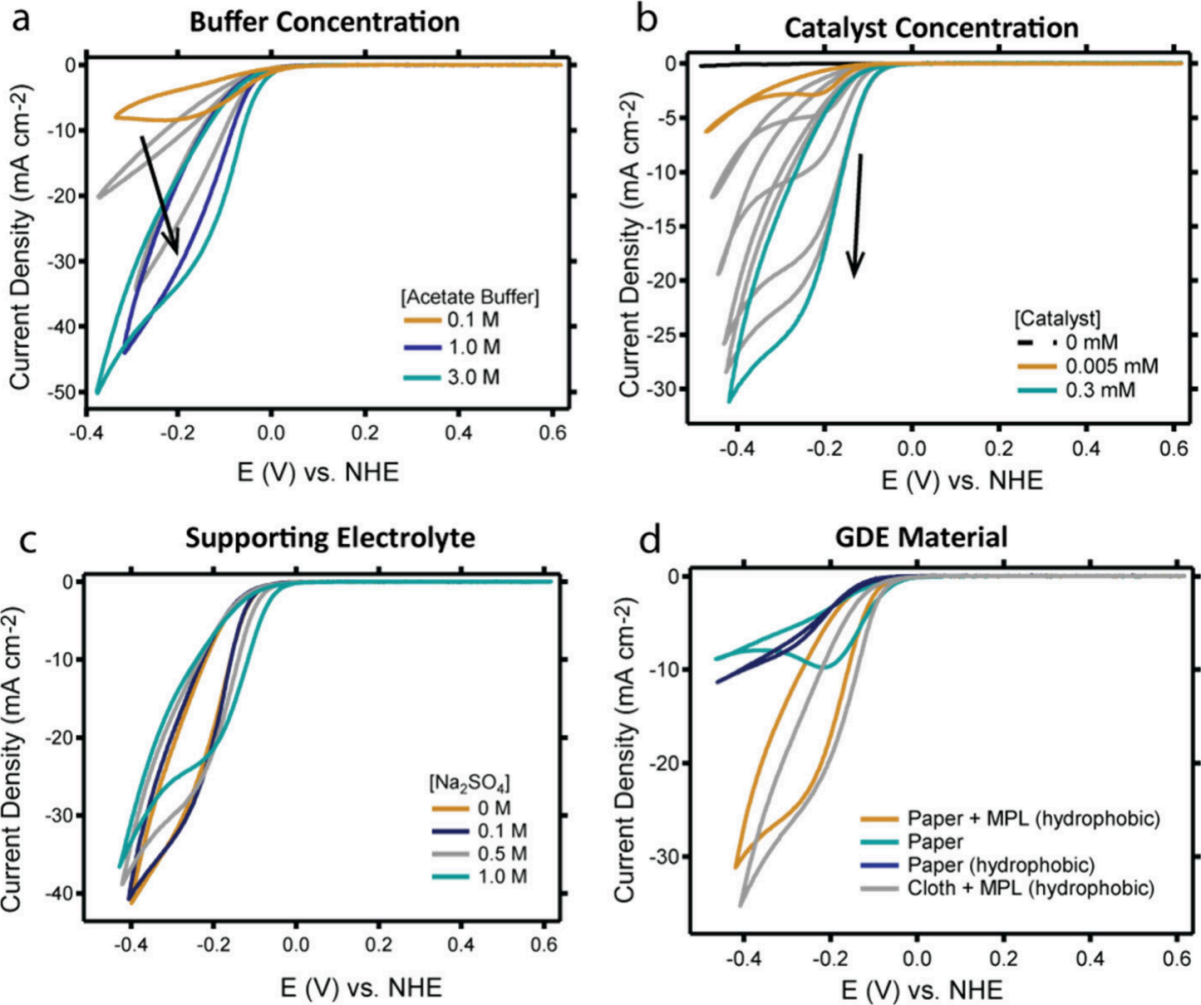
CV measurements of the ORR activity of homogeneous Cu­(tmpa) in a GDE setup with (a) increasing buffer concentration from 0.1 M (orange) to 0.2–0.5 (grey), 1.0 M (blue), and to 3.0 M (light blue), (b) increasing catalyst concentration from 0.005 mM (orange) to 0.01–0.05–0.1–0.2 mM (grey) to 0.3 mM (light blue), (c) increasing concentration of Na_2_SO_4_ as supporting electrolyte from 0 M (orange) to 0.1 M (dark blue), 0.5 M (grey), and 1.0 M (light blue), and (d) varying GDE materials. Conditions: H23C8 GDE material (except (d)), 0.3 mM Cu­(tmpa) (except (b)), 3.0 M AB, pH 4.8 (except (a)), 100 mV/s scan rate, and full iR compensation. The black arrow indicates the progression of the current density as a function of concentration.

CV measurements were recorded in acetate buffers of different concentrations ranging from 0.1 to 3 M (Figure 3a). A scan rate of 100 mV/s was selected, allowing for comparison with stationary experiments, which were performed at the same scan rate of 100 mV/s. In studies simultaneous with this work, we have shown that Cu­(tmpa) remains highly active in this buffer and is able to generate H_2_O_2_ solutions with a slightly higher selectivity and stability.^58^ Moreover, in contrast to commonly used phosphate buffers, the use of acetate buffers can lower the *iR* substantially, and a much lower resistance is found at higher buffer concentrations (see Supporting Information Section 2), albeit with a *Z*′ that is still quite high (Figures S1 and S2). It is evident that a higher buffer concentration results in a larger catalytic current, and therefore, the highest buffer concentration of 3 M was utilized in all further CV measurements (Figure 3a). The strong effect of the buffer concentration also indicates that catalysis is limited by the availability of protons in solution. Even at a high buffer concentration of 3 M, the catalytic CV has a clear shoulder in the forward scan and exhibits hysteresis. We assume this shoulder originates from the depletion of protons and a switch in proton source from acetic acid to H_2_O, as reported before for RDE measurements.^58^ The local accumulation of OH^–^ during catalysis and the induction of a concentration-dependent overpotential, most pronounced in the backward scan, will cause hysteresis in the CV measurement.^75^ The above observations are further supported by a decrease in the catalytic current in consecutive CV scans and the fact that this decrease can be counteracted by stirring of the solution to induce transport of OH^–^ to the bulk (Figure S3).

CV measurements were recorded in the presence of different concentrations of Cu­(tmpa) catalyst. Whereas in RDE measurements the Cu­(tmpa) concentration can be lowered to the micromolar range without causing any change in catalytic current,^58^ Figure 3b shows that higher catalyst concentrations correlate to higher catalytic current in a GDE setup. In presence of 5 μM catalyst, the shoulder of the catalytic current reaches approximately 3 mA/cm^2^, while the catalytic current increases to 25 mA/cm^2^ at the same potential in the presence of 0.3 mM catalyst. To maintain a high catalytic current and to compare this work to previous electrochemical studies of Cu­(tmpa), a concentration of 0.3 mM was chosen for further experiments.

We also investigated whether catalysis would benefit from the addition of supporting electrolyte (Figure 3c). CVs were measured in 3 M acetate buffer and additional Na_2_SO_4_ up to 1 M. The addition of Na_2_SO_4_ lowers the catalytic current, even though it increases the conductivity of the electrolyte (Supporting Information Section 2). We hypothesize that this effect originates from the sulfate from the Na_2_SO_4_ electrolyte coordinating to the Cu site of the catalyst, thereby inhibiting O_2_ binding to Cu­(tmpa). Due to these observations, further experiments were conducted in the absence of additional Na_2_SO_4_.

Finally, we benchmarked different types of GDEs at the same buffer concentration (3 M) and catalyst concentration (0.3 mM) (see Table S1 for specifications). We strategically selected four GDEs to investigate the impact of a hydrophobic treatment and/or a microporous layer (MPL) as well as the use of carbon paper versus carbon cloth. Figure 3d shows that the type of GDE has a large effect on the catalytic current that is measured. The two highest catalytic currents were obtained for a GDE with a MPL. It is known from heterogeneous catalysis that the MPL increases the electrical contact between the GDL and the catalyst layer;^76^ hence, we assume a MPL will have a similar effect and increases the contact between the GDE and the dissolved catalyst. In addition, an MPL enhances the catalytic performance, in terms of current density at low overpotential, because it is beneficial for water drainage and prevents flooding.^76–78^ Whether or not the GDE underwent hydrophobic treatment seems to have a small effect. The catalytic current measured on carbon paper GDEs without MPL is only slightly lower compared with the GDE with hydrophobic treatment. However, hydrophobic treatment of the electrode is beneficial to prevent the breakthrough of the electrolyte during catalysis. Figure 3d shows that similar results are obtained for carbon cloth and carbon paper. Due to the lower costs of carbon paper, a carbon paper GDE was selected for further experiments.

### H_2_O_2_ Generation in a GDE Cell

After the measurements conducted in the small GDE cell, a high buffer and catalyst concentration in combination with the carbon paper GDL with MPL and hydrophobic treatment (Freudenberg H23C8) were selected as conditions to generate H_2_O_2_ in the larger GDE flow cell. As to minimize excessive amounts of electrolyte salts, the flow electrolysis setup uses 160 mL of electrolyte, and acetate buffer of 1 M instead of 3 M was used. This has a minor effect on the catalytic current, since the difference between CV measurements of the ORR activity in the presence of 1 and 3 M buffer is fairly small (Figures 2a and S4). After assembly of the flow cell, LSV and CV measurements were recorded both in the presence and in the absence of catalyst to make sure that the setup functioned correctly. Thereafter, oxygen reduction electrolysis was carried out during four consecutive hours at a current density of −10.3 mA/cm^2^ at a potential of approximately −0.2 V vs NHE (Supporting Information Section 4.1), while FE_H2O2_ was periodically determined by taking aliquots of the catholyte from the electrolyte reservoir. At this potential, the rate constant of the two-electron ORR by Cu­(tmpa) was determined to be more than 10 times higher than that of the HPRR, which allows high selectivity for H_2_O_2_ as long as the local concentration of O_2_ remains high compared to the concentration of H_2_O_2_.^58^

We anticipated that GDEs significantly increase the availability of O_2_ compared to a RDE configuration, and therefore, a GDE setup should be beneficial to obtain a high FE_H2O2_. However, initial experiments obtained a stable but low FE_H2O2_ of 5% (Figure S6). This FE value is substantially smaller than the 50% previously obtained in the RDE setup during electrolysis.^58^ A large decrease in FE_H2O2_ moving from an idealized rotating (ring-)­disk electrode R­(R)­DE system to a GDE setup is unexpected but not uncommon. A similar observation was previously ascribed to the increased residence time of H_2_O_2_ in the vicinity of the GDE electrode, compared to the time spent close to the surface of an R­(R)­DE.^79^

### Selectivity of H_2_O_2_ Generation

To further understand the relatively low ORR selectivity in a GDE setup, FE_H2O2_ was determined in 6 consecutive electrolysis measurements of 30 min with different current densities between −5 mA/cm^2^ and −26 mA/cm^2^ in the flow cell GDE. It was shown that variation of the liquid flow rate in the range of 30–40 mL/min and gas flow rates in the range of 30–40 mL/min do not significantly affect the Faradaic yields (Figure S7). Interestingly, FE_H2O2_ strongly depends on the applied current density and increases when the current density becomes larger (Figure 4 and Figure S8). The current density of −26 mA/cm^2^ reaches a FE_H2O2_ of more than 50%, while the FE_H2O2_ is only a few percent when −5 mA/cm^2^ is applied. Since the experiments were conducted in a randomized order, we can rule out that the observed trend is caused by any electrode modifications or deposition of the catalyst onto the electrode that might occur over time. Control experiments showed that the measured FE_H2O2_ is not strongly dependent on the flow of O_2_ or electrolyte in the system (Supporting Information Section 4.2), and thus, mass transport of O_2_ towards the catalyst or H_2_O_2_ transport away from the electrode seem to have some effect on the current but a minor effect on the FE_H2O2_. In addition, the H_2_O_2_ concentration is stable over multiple hours in acetate buffer, and control measurements rule out that the setup breaks down any generated H_2_O_2_ when no potential is applied (Supporting Information Section 7.4, Figures S16 and S17).

**Figure 4:**
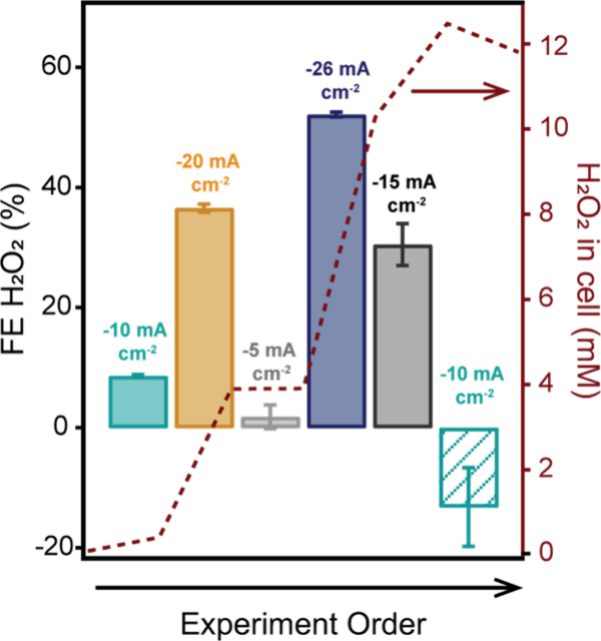
Faradaic efficiency (FE) towards H_2_O_2_ obtained from electrolysis measurements at different current densities (left axis) and the concentration of H_2_O_2_ present in the electrochemical cell during the measurements (right axis) obtained in a GDE setup in the presence of homogeneous Cu­(tmpa). The order of the applied current densities is indicated by the arrow below the graph. The measurement at −10 mA/cm^2^ was carried out twice.

It is likely that the pH plays an important role in the selectivity of the ORR. The buffer reaches its capacity at high current densities, and the local pH increases. This was namely observed in the CVs were recorded in the small GDE cell (Figure 3a). Since Cu­(tmpa) is able to catalyze both the two-electron reduction of oxygen and the further two-electron reduction of H_2_O_2_, the selectivity of Cu­(tmpa) depends on the ratio between the rate of the HPRR and the ORR.^41,58^ The effect of the pH on the HPRR has not been investigated, while the ORR rate has been previously investigated in CV measurements, showing that the activity decreases in alkaline conditions.^58,80^ Examination of the ORR activity of Cu­(tmpa) in the small GDE setup at pH 9 shows that the catalytic current largely decreases under these conditions (Figure S5). Moreover, the catalytic ORR current of the GDE itself increases under alkaline conditions, but Cu­(tmpa) remains more active. The GDE itself is not active for the HPRR, while the HPRR activity of Cu­(tmpa) is drastically lowered moving from pH 5 to pH 9. Therefore, we hypothesize that the local increase of the pH at high current densities will suppress the HPRR activity and boost the FE_H2O2_.

The bulk electrolysis measurement of −10 mA/cm^2^ in Figure 4 was conducted twice, obtaining different FE_H2O2_ values. In the first measurement, the FE_H2O2_ was less than 10%, in line with the results from a similar experiment in Figure S6, while the FE_H2O2_ value became negative in the second experiment. We anticipate that, in this specific experiment, the concentration of H_2_O_2_ in the catholyte reached such a high concentration that the HPRR took over. Albeit in an stationary setup the observed rate constant of the ORR is more than 10 times larger than for the HPRR,^41^ it is anticipated that in a GDE setup different mechanisms hold and high H_2_O_2_ concentrations will limit its net generation.

### Catalyst Heterogenization

Up to this point, Cu­(tmpa) was present as a dissolved species in the electrolyte solution. Nevertheless, the separation of the catalyst from the H_2_O_2_ solutions is favored in practical reactor design. Moreover, Cu­(tmpa) slowly breaks down H_2_O_2_ in the absence of applied current.^58^ It is therefore relevant to heterogenize Cu­(tmpa) onto the electrode surface. Studies with heterogenized molecular catalysts were previously conducted using Cu­(tmpa) and related copper catalysts on nickel oxide,^81^ carbon nanotubes,^82^ and GC electrodes,^83^ but never using a GDE. To heterogenize the catalyst, an ink of Nafion, carbon black, and Cu­(tmpa) was prepared and deposited onto the electrode. In case of the small GDE cell, the ink was dropcasted to achieve a catalyst loading of 0.59 mg/cm^2^, and in case of the GDE flow cell, the ink was spray coated onto the electrode surface to achieve a comparable catalyst loading of 0.66 mg/cm^2^ (see Supporting Information Section 1.6 for details and Figure 6e for a representation).

CV measurements in the small GDE cell were recorded to compare the properties of the homogeneous catalyst to those of its heterogenized counterpart (see Supporting Information Section 5 and Figure 5). Measurements of heterogenized Cu­(tmpa) in the presence of Ar show a clear redox couple, confirming the presence and redox activity of the copper sites in the ink (Figure 5a). In line with this, the deposition of double the amount of ink resulted in an increase in the electrochemically accessible copper sites (Figure 5b). Interestingly, the half-wave potential (*E*_1/2_) of the Cu^II^/Cu^I^ redox couple of the dropcasted catalyst is −99 mV vs NHE, while the *E*_1/2_ of homogeneous Cu­(tmpa) in solution is −212 mV vs NHE for a GDE and −147 mV vs NHE for a GC work electrode (Supporting Information Section 6). *E*_1/2_ is thus more negative when the working electrode is a GDE instead of a RDE electrode, and in turn, heterogenization of the catalyst onto the GDE shifts *E*_1/2_ to a more positive potential (Figure S10). Previously, it was discussed that the reduction of Cu^II^ to Cu^I^ is the potential determining step in the oxygen reduction reaction,^41^ which directly scales to log *j*_cat_.^43^ Such a redox couple shift upon heterogenization of a molecular catalyst was not previously reported for Cu­(tmpa) or any other molecular catalyst but can be an explanation for the remarkably small overpotential of Cu­(tmpa) when it was heterogenized on the surface of a GC electrode using a similar ink.^83^

**Figure 5:**
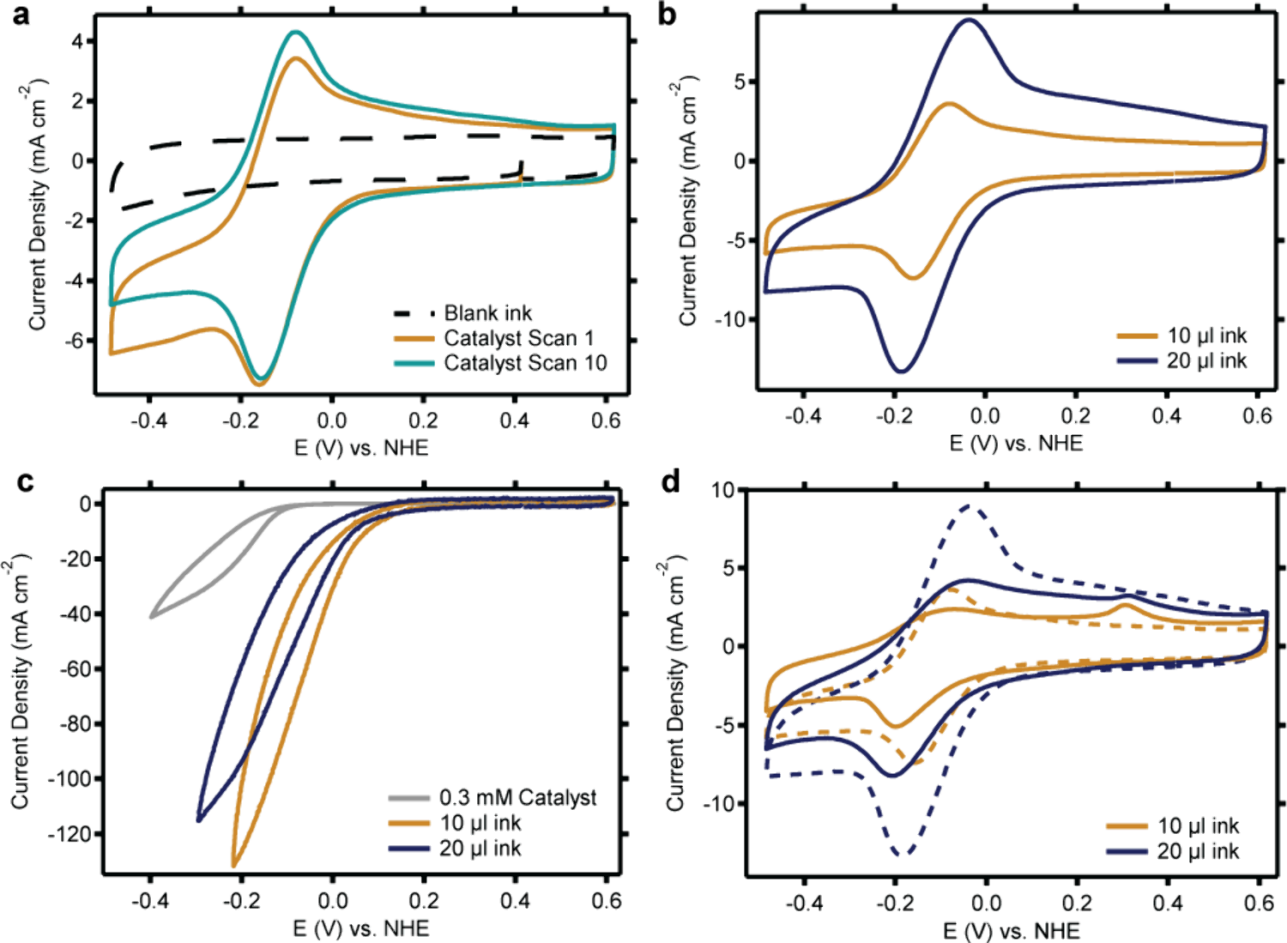
CV measurements were recorded in the small GDE cell to investigate various forms of heterogenized Cu­(tmpa) catalyst in comparison to the homogeneous catalyst. (a) Redox couple of dropcasted Cu­(tmpa) recorded during 10 consecutive scans prepared from dropcasting 10 μL of catalyst ink. (b) Redox couple of Cu­(tmpa) recorded on a GDE prepared from dropcasting 10 μL (orange) or 20 μL (dark blue) of catalyst ink. (c) Catalytic activity of heterogenized Cu­(tmpa) recorded on a GDE prepared form dropcasting 10 μL (orange) or 20 μL (dark blue) of catalyst ink compared to measurements of 0.3 mM of the homogeneous catalyst in solution. (d) Redox couple of Cu­(tmpa) recorded on a GDE prepared from dropcasting 10 μL (orange) or 20 μL (dark blue) of catalyst ink before (dashed line) and after (solid line) recording 10 CV scans in the presence of an O_2_ flow field. Conditions: 0.59 or 1.18 mg/cm^2^ catalyst loading; 3.0 M acetate buffer, pH 4.8; Ar or O_2_ flow field; H23C8 GDE; 100 mV/s scan rate; full iR compensation.

In the presence of O_2_, the catalytic wave of the heterogenized catalyst is shifted to a more positive potential compared to that of the homogeneous catalyst in solution. This shift is in line with the shift of almost 50 mV of the Cu^II^/Cu^I^ redox couple and indicates that reduction of the Cu^II^ site remains an essential descriptor for the potential at which Cu­(tmpa) can generate oxygen.^43^ The current of the heterogenized catalyst is larger than that of the homogeneous catalyst (Figure 5). This is the result of all Cu sites being in close contact with the electrode interface in the case of heterogenization, while in case of a homogeneous catalysts, most Cu sites are homogeneously spread out in the electrolyte solution with the far majority of Cu species too far away from the electrode to undergo electrochemical reactions. As a result, heterogenization results in a higher catalyst availability, even though less catalyst is used in total. Interestingly, the catalytic current does not depend on the amount of ink that is dropcasted, even though the number of accessible copper sites increases. Most likely, the catalyst loading in this case is so high that the catalytic current is independent of the catalyst loading.

Next, the heterogenized catalyst was used to generate H_2_O_2_ in the GDE flow cell (Figure 6). In the case of the larger electrode employed, the catalyst was spray coated instead of dropcasted. In line with the trends of the small GDE cell, the heterogenized spray coated catalyst reaches a higher current density at a lower overpotential compared to the homogeneous catalyst in CV measurements (Figure 6c). Next, the catalytic activity of the homogeneous catalyst and the heterogenized catalyst were compared in electrolysis measurements at a current density of −10 mA/cm^2^ for 1 h and at a current density of −20 mA/cm^2^ for three more hours (Figure 6d). In both cases, the same trend is observed: a larger current density results in a higher FE_H2O2_ (Figure 6a). At an applied current density of −10 mA/cm^2^, the FE does not exceed 10%, whereas at −20 mA/cm^2^, the FE increases to almost 30%. For the heterogeneous catalyst, the FE_H2O2_ values slightly increase over the course of 3 h, while there is no such trend in the case of the homogeneous catalyst. The latter one appears to drop in selectivity, which may be related to disproportionation of peroxide in the presence of substantial concentrations of Cu­(tmpa) in solution,and/or degradation of Cu­(tmpa), leading to formation of copper deposits.

**Figure 6:**
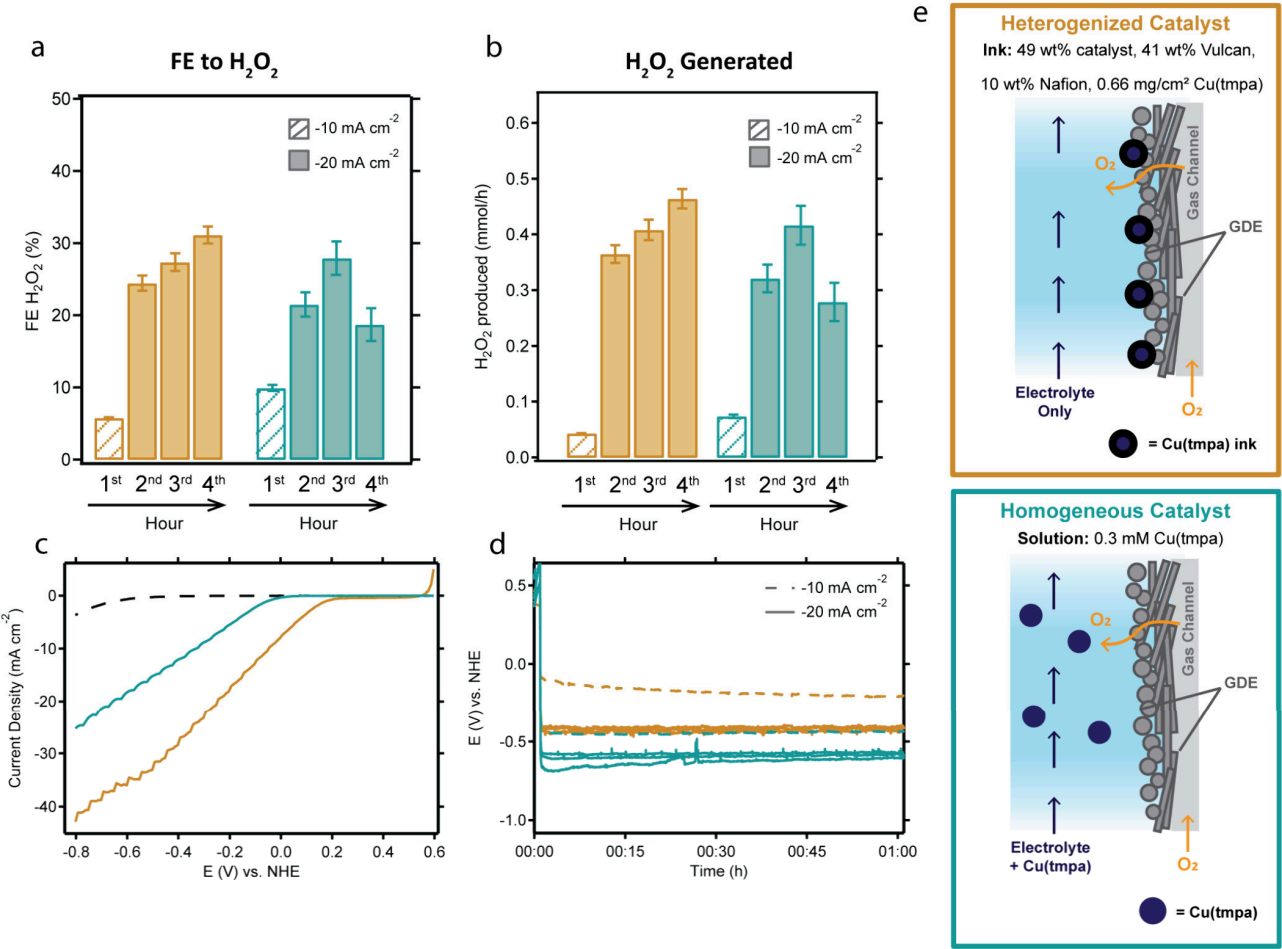
An overview of the measurements comparing the heterogenized Cu­(tmpa) catalyst (orange, spray-coated) to the homogeneous Cu­(tmpa) catalyst in solution (light blue) for generation of H_2_O_2_ during electrolysis in the GDE flow cell. The amount of H_2_O_2_ that is generated at a current density of −10 mA/cm2 (striped) and −20 mA/cm^2^ (solid) are shown (a) in terms of the FE towards H_2_O_2_ and (b) in terms of H_2_O_2_ produced. (c) LSV measurements of the homogeneous catalyst (light blue) and heterogeneous catalyst (orange), showing the earlier onset for the heterogeneous case. (d) Voltammogram of the measured potential by application of −10 mA/cm^2^ (dashed lines) and −20 mA/cm^2^ (solid lines) for the homogeneous (orange) and heterogeneous (light blue) cases. Multiple duplicates are shown for the −20 mA/cm^2^ experiment. (e) Schematic representation of the heterogenized catalyst on the GDE surface prepared from an ink (top) and the homogeneous catalyst in solution. Conditions: 0.3 mM Cu­(tmpa) in solution or 0.66 mg/cm^2^ surface loading, 1.0 M acetate buffer, pH 4.8, an O_2_ flow field, an H23C8 GDE, a 100 mV/s scan rate, and no iR compensation.

### Catalyst and GDE Structure during Catalysis

We analyzed the structure of Cu­(tmpa) during catalysis as well as any changes in the GDE electrode, which becomes substantially more active over time (Figure S11). In previous work, the stability of Cu­(tmpa) during ORR electrolysis in an RDE setup was thoroughly investigated.^58^ This work showed that in the presence of large catalyst concentrations a copper-based deposit would form on the electrode surface, although with a lower catalytic activity, but ultimately being detrimental to the FE_H2O2_.

CV measurements recorded in the small GDE cell in the presence of homogeneous Cu­(tmpa) over a time interval of 2 h did not show any changes in the measured catalytic current (Figure S12a). When 25 consecutive ORR CV scans were recorded, a slight increase in the catalytic current was observed. Recording a CV of this electrode in the absence of catalyst ruled out that this increase was caused by formation of catalytically active deposits on the electrode (Supporting Information Section 7.2). However, CV measurements in absence of catalysts showed that the catalytic current of the bare GDE increases during consecutive scans, especially when the lower potential limit of the CV scan is sufficiently negative. The GDE material itself will thus be slightly modified when reductive potentials are applied (Supporting Information Section 7.1), which is in line with previously reported trends.^84,85^

Next, the stability of the homogeneous Cu­(tmpa) catalyst during electrolysis was further investigated. To do so, CVs were recorded right before and after a 4 h chronopotentiometry experiment at an applied current of −10 mA/cm^2^. CVs recorded afterwards indicated that the catalytic current increased during electrolysis. In addition, a metallic copper species was deposited onto the electrode, as implied by the occurrence of an extra oxidation event above 0.2 V vs NHE (Figure S6). In line with this, UV-vis spectra of the electrolyte solution indicated that the concentration of Cu­(tmpa) in the electrolyte was substantially lowered over the time course of 4 h (Figure S13a). However, the decrease in catalyst concentration did not result in a loss of catalytic activity in the GDE flow cell.

For the dropcasted heterogenized catalyst, CV measurements in the small GDE cell indicated that the catalyst gets partially broken down during catalysis of the ORR. Extra oxidation events were observed, and the Cu­(tmpa) redox couple was slightly decreased after catalysis (Figure 5d). The stability of the spray coated heterogenized catalysts before and after electrolysis in the GDE flow cell was further analyzed by UV-vis and SEM-EDX (Figures S14 and S15). No Cu­(tmpa) was detected in the electrolyte by UV-vis; although if all catalyst would have leached out of the ink, this would have been clearly visible (Figure S13b). In addition, SEM-EDX analysis of the GDE did not indicate the loss of any copper from the surface during catalysis (Supporting Information Section 7.3).

### GDE vs RDE

The results of the GDE can be compared to the data obtained with an RDE configuration in terms of catalyst loading, current density, selectivity, and H_2_O_2_ generation (Figure 7). In an RDE setup, the catalyst concentration has a big effect on the selectivity of the ORR.^58^ The FE_H2O2_ can be substantially increased when the catalyst concentration is lowered to the micromolar range, as the local concentration of O_2_ remains high and the HPRR gets suppressed. Moreover, the current response in the RDE CV measurements is independent of the catalyst concentration up to the micromolar range, as catalysis is severely limited by O_2_ mass transport. This situation is completely different in the GDE setup. This work shows that the catalytic current in the GDE system strongly depends on the catalyst concentration, and consequently, a 60 times higher catalyst concentration was chosen for H_2_O_2_ generation. Fortunately, heterogenization of the catalyst onto the electrode surface minimizes the total amount of catalysts needed and provides stabilization.

**Figure 7:**
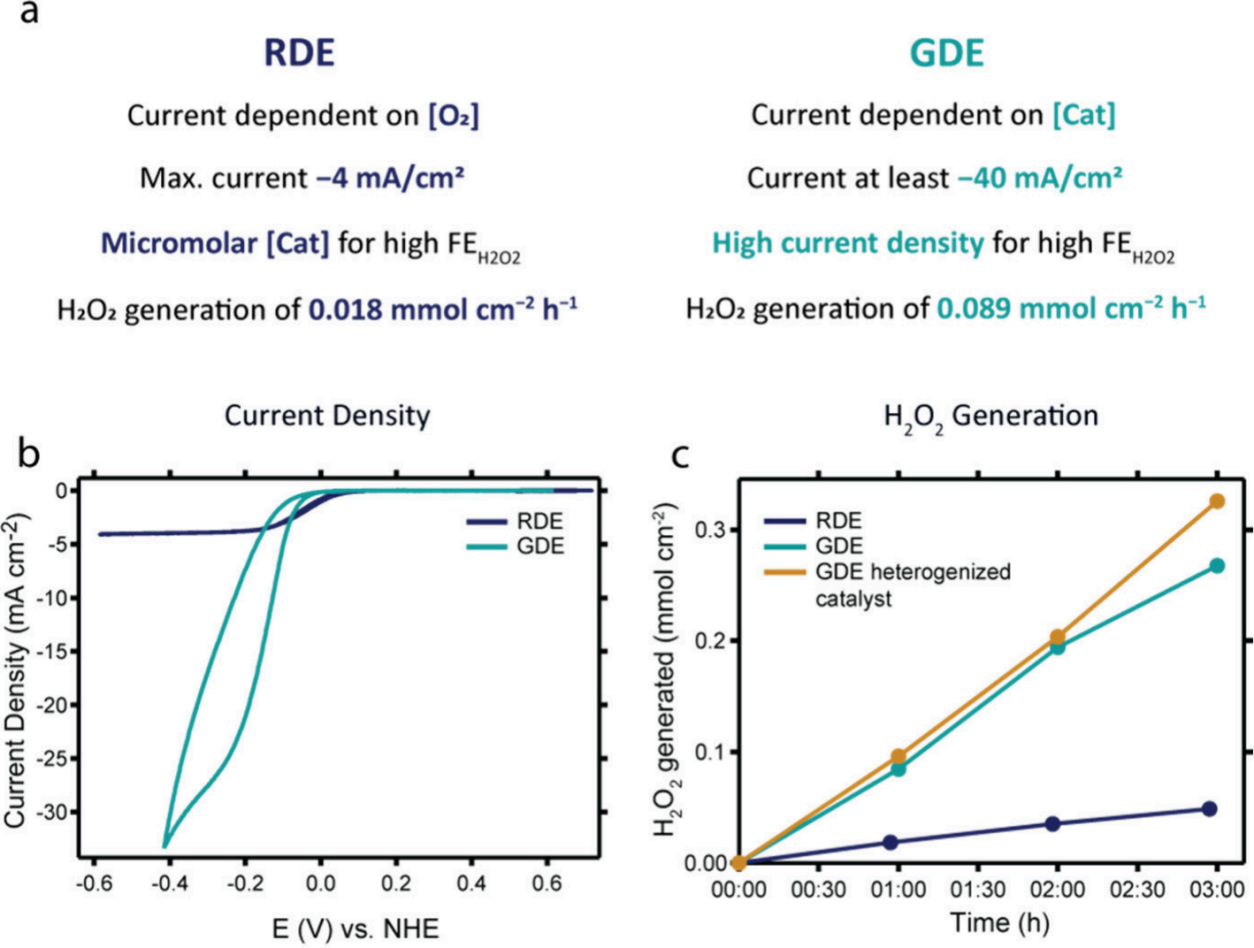
(a) Comparison of the results obtained in an RDE setup and a GDE setup as discussed in the text. (b) CV measurements of homogeneous Cu­(tmpa) in the RDE setup (dark blue) and small GDE cell (light blue) in the presence of O_2_. (c) H_2_O_2_ generated during electrolysis in different setups: RDE (dark blue), GDE flow cell + homogeneous catalyst (GDE hom, light blue), and GDE flow cell with spray-coated catalyst (GDE het, orange). Conditions for (b): 1.0 M actetate buffer (RDE) and 3.0 M (GDE), pH 4.8, 0.3 mM Cu­(tmpa), 50 mV/s, O_2_ atm/flow field, GC disk or H23C8 GDE, and full iR compensation GDE. Conditions for (c): for RDE, 0.1 M acetate buffer, pH 4.8, 0.005 mM Cu­(tmpa), 1600 rpm, O_2_ atmosphere, and GC electrode; for GDE homogeneous catalyst, 1.0 M acetate buffer, pH 4.8, H23C8 GDE, −20 mA/cm^2^, and 0.3 mM Cu­(tmpa); for GDE heterogeneous catalyst (orange), 1.0 M acetate buffer, pH 4.8, H23C8 GDE, −20 mA/cm^2^, and 0.66 mg/cm^2^ Cu­(tmpa). Data for the RDE measurements in (b) are obtained from ref 58.

The current density is increased almost 10 times in the GDE configuration compared to the RDE setup (Figure 7b, Figure S18, and Supporting Information Section 8). In the RDE setup, a limiting plateau current of approximately −4 mA/cm^2^ is reached, while current densities of up to almost −40 mA/cm^2^ are obtained in the GDE cells. The current density in the GDE configuration is further improved by increasing the catalyst concentration or heterogenization of the catalyst onto the GDE surface. Interestingly, the onset potential of the ORR is more positive for the RDE setup than for the GDE setup. This is in line with the potential shift found for the Cu­(tmpa) redox couple and indicates that the onset potential of the catalytic wave is strongly correlated to the reduction of the catalyst (Figure 5).

The relation between the current density and FE_H2O2_ found in the GDE setup is counterintuitive with respect to the results of the RDE measurements.^58^ In RDE measurements, overreduction of the generated H_2_O_2_ to H_2_O would take place at a more negative potential, and therefore, a higher FE_H2O2_ is obtained close to the catalytic onset potential. Contrasting this, in the GDE setup, a higher selectivity was found at lower potentials and higher current densities. We attribute this discrepancy to the effect of the increased local pH in the GDE setup that originates from the large current densities. We hypothesized that at a high pH the HPRR activity is suppressed, thereby favoring the two-electron ORR.

In an RDE setup, Cu­(tmpa) can generate H_2_O_2_ in a 0.1 M acetate buffer with a good FE_H2O2_ of more than 50% during multiple hours.^58^ This corresponds to H_2_O_2_ generation with an average rate of 0.018 mmol cm^–2^ h^–1^. In the GDE flow cell, the rate of H_2_O_2_ generation reaches an average of 0.089 mmol cm^–2^ h^–1^ in the presence of Cu­(tmpa) as a homogeneous catalyst at −20 mA/cm^2^, whereas the heterogenized catalyst produces 0.11 mmol cm^–2^ h^–1^ under the same conditions. This indicates an increase in the rate of H_2_O_2_ generation of more than 5× times, as shown in Figure 7c. In terms of absolute quantities, the GDE setup generates 0.33 mmol/h H_2_O_2_ in the presence of the homogeneous catalyst and 0.41 mmol/h for the heterogenized catalyst, which is 100 times more H_2_O_2_ than in the RDE setup (Supporting Information Section 8). In the RDE measurements of Cu­(tmpa), a H_2_O_2_ solution of 0.6 mM could be generated without a loss in the FE_H2O2_.^58^ In contrast, the H_2_O_2_ concentration in the GDE flow cell reached more than 12 mM, but it resulted in a drop in the FE_H2O2_. However, a H_2_O_2_ concentration of only a few micromolar is sufficiently high to use in water treatment,^86–88^ and therefore, the H_2_O_2_ electrolyte solution obtained in this work is suitable for applications.

## Conclusion

For the first time, a molecular catalyst has been studied in the liquid phase with GDE electrodes, and the electrochemical selectivity, short-term stability, and activity of a homogeneous catalyst configuration for hydrogen peroxide generation via the two-electron reduction of oxygen have been reported. In general, it is shown that the reactivity of Cu­(tmpa) is similar, but not identical, to that observed under stationary conditions and rotating disk configurations. The catalytic activity of the molecular copper-based Cu­(tmpa) catalyst was studied in two different GDE setups. A small GDE cell was used to identify that a high buffer capacity is required to maintain high activity, and a high catalyst concentration increases the catalytic current. Under these conditions, the current density for the ORR can be increased almost 10-fold in a GDE configuration compared with a conventional RDE setup. Hydrogen peroxide was generated during multiple hours of constant current electrolysis in a GDE flow cell. From these measurements, it is evident that Cu­(tmpa) is able to generate H_2_O_2_ with a stable activity of 26 mA/cm^2^ and a FE_H2O2_ that can be increased up to 50%. These are the highest current densities recorded for a truly homogeneous electrocatalyst for the ORR thus far.

In light of potential applications, we have shown that Cu­(tmpa) can be heterogenized onto the GDE surface using a catalytic ink. This heterogenization reduces the amount of catalyst that is needed for measurements and also drastically lowers the onset potential of the ORR, while the FE_H2O2_ remains similar to that of the homogeneous catalyst in solution. When Cu­(tmpa) is heterogenized onto the GDE surface, H_2_O_2_ can be generated at a rate of 0.11 mmol cm^–2^ h^–1^ at a current density of −20 mA/cm^2^. It should be noted that the Cu­(tmpa) catalyst, especially in homogeneous form, suffers from stability problems under the applied conditions in the GDE cell, and we therefore encourage future research to explore the stability of molecular catalysts for H_2_O_2_ generation in a GDE setup. Overall, this work not only shows the practical potential of molecular ORR catalysts for the electrochemical generation of H_2_O_2_ in industrially relevant reactor configurations but also highlights the usefulness of GDE platforms in molecular electrocatalysis.

## Supplementary Material



## Notes

Experimental information, SEM/EDX measurements, electrochemical experiments, and UV-vis spectra (PDF)
