# Supplementary material for: H2O2 Synthesis with Molecular Electrocatalysts Enables Substantial Current Densities in a Flow Cell Using Gas Diffusion Electrodes
Source: ACS Electrochem. 2025 Oct 8;1(11):2326–37. doi: 10.1021/acselectrochem.5c00179 (PMC12598710; doi:10.1021/acselectrochem.5c00179)
Supplement: Supplementary file 1 [file ec5c00179_si_001.pdf]

H_2O_2 Synthesis with Molecular Electrocatalysts enables Substantial Current Densities in a Flow Cell using Gas Diffusion Electrodes

Supporting Information

Phebe H. van Langevelde[a], Nathalie E.G. Ligthart[b], Pim G.J. van Duren[a], David A. Vermaas[b*], and Dennis G.H. Hetterscheid[a*]

[a]Leiden Institute of Chemistry, Leiden University, 2300 RA, Leiden, The Netherlands

[b]Department of Chemical Engineering, Delft University of Technology, 2629 HZ, Delft, The Netherlands

Email: d.g.h.hetterscheid@chem.leidenuniv.nl, d.a.vermaas@tudelft.nl

Contents

1. Experimental 3

1.1 General 3

1.2 RDE setup 3

1.3 Small GDE setup 3

1.4 GDE flow cell 3

1.5 GDE materials 4

1.6 GDE with drop casted ink 4

1.7 GDE with spray-coated ink 5

1.8 Specifications of electrochemistry setups 5

1.9 Determination of the H_2O_2 concentration 5

2. *iR* Compensation 6

3. CV Measurements in the Small GDE Cell 7

3.1 Effect of pH 8

4. GDE Flow Cell 8

4.1 4-hour electrolysis 8

4.2 Effect of liquid and gas flow rates 10

4.3 Effect of current density 11

5. Measurements with Heterogenized Catalyst 12

6. Cu(tmpa) Redox Couple 12

7. Stability Investigations 12

7.1 Stability of the GDE 12

7.3 Catalyst stability in electrolysis 14

7.4 Stability of H_2O_2 solutions 17

8. H_2O_2 Generation in Different Setups 18

9. References 18

1. Experimental

1.1 General

All electrolyte solutions were prepared from high purity salts and acids, Na_2SO_4 (99.99% Suprapur, Merck), CH_3COOH (TraceSELECT ≥99.0%, Honeywell Fluka), $NaCH_3COO$ (≥99.999% TraceSELECT, Honeywell Fluka) and Milli-Q. For large-scale H_2O_2 generation in the flow cell GDE, 1.0 M acetate buffer was prepared from CH_3COOH (>99%, Alfa Aesar), and $NaCH_3COO$ (>99%, Thermo Scientific) and de-ionized water. The pH of the electrolyte solutions was measured with a HI 4222 pH meter from Hannah Instruments, which was calibrated regularly. The O_2 and Ar gasses used in electrochemical measurements were supplied by Linde. UV-vis spectra were recorded on a Varian Cary 50 spectrophotometer from Agilent. For preparation of the catalyst inks Nafion D-521 dispersion (5 wt%, Alfa Aesar) and Carbon Black (Vulcan XC 72, Fuell Cell Store) were used. Scanning electron microscopy (SEM) images were obtained using a Thermo Scientific Apreo scanning electron microscope operating under a high vacuum. An accelerating voltage of 15–20 kV with a 0.1–1.6 nA probe current was used for data collection. For energy dispersive X-ray (EDX) measurements an UltraDry energy dispersive X-ray detector was used. [Cu(tmpa)MeCN]OTf_2 (tmpa = tris(2pyridylmethyl)amine) was synthesized as previously reported by our group.[1] All our synthesis experiments are carried out in the same manner as what has been described in this reference and are cross checked in terms of UV-vis, EA and CV for every new synthesis.

1.2 RDE setup

All RRDE measurements are carried out using an Autolab PGSTAT 12 potentiostat in combination with a Pine Instruments rotator, a ChangeDisk RRDE electrode (E6R1PK) from Pine Instruments with a PEEK shroud, a 5 mm GC disk (A = 0.196 cm^2) and a Pt ring, in an custom-build three electrode electrochemical cell with a minimal volume of 40 ml. All RDE data and detailed procedures have been reported previously.[1,2]

1.3 Small GDE setup

The small GDE cell configuration is commercially available via www.gde-cell.com. The GDE cell consists of two parts made from PEEK: a top part for the electrolyte and a bottom part with a gas flow field and two gold pins for connection of the working electrode (GDE). The top part of the cel was filled with 15 ml of electrolyte and regularly cleaned with the $KMnO_4$ procedure described above, and boiled in Milli-Q water for 30 minutes prior to every measurement.

Before every measurement a piece of a GDE was cut and secured between the bottom and the top part of the cell. The resulting contact area between the GDE and the electrolyte solution is a circle with a diameter of 3 mm (A= 0.071 cm^2). As a reference electrode, a hydroflex electrode from Gaskatel or a single junction Ag/AgCl electrode (3M KCl) from Metrohm were used. The equilibrium potential of these electrodes was regularly measured versus a RHE electrode to confirm the accuracy of the measurements. A gold wire was used as counter electrode and placed in a separate, closed glass tube connected to the main cell compartment with a glass frit. During experiments, humidified O_2 gas was flowed through the bottom compartment of the GDL cell. A flow meter was connected to the setup, but the measurements were not sensitive to the flow rate of O_2.

1.4 GDE flow cell

A detailed description of the GDE flow cell was reported previously by one of our groups.[3] The electrolysis cell was made out of custom-made poly methyl methacrylate (PMMA) plates. These PMMA plates were sealed against each other using 0.5 mm thick silicone gaskets (Eriks). The cathode GDE was placed in front of a PMMA plate with a hole to expose 3.8 cm^2 of its surface to the electrolyte. The other side of the GDE was exposed to a gas compartment channel and connected to copper tape (Conrad) as a current collector. A Ag/AgCl micro-reference electrode (Leak-free Ag/AgCl LF-1-45, Alvatek) was placed into the PMMA plate close to the GDE.

As the anode, a titanium disk coated with an IrO_2 oxygen evolution catalyst (Magneto Special Anodes) was used. The cathode and anode compartment were separated by a Nafion N-117 (DuPont) proton exchange membrane (PEM). This Nafion membrane was boiled in de-ionized water for 30 minutes at first and stored in acetate buffer in between measurements.

Both the catholyte and anolyte compartments comprised 80 ml electrolyte. This electrolyte was pumped through the cell by a peristaltic pump (Masterflex L/S peristaltic pump, Cole Parmer) at a pump rate of 30 RPM. In addition, the liquid back-pressure of both the catholyte and analyte channel were controlled with electronic control valves (P-502C-6K0R, Bronkhorst). The O_2 flow rate of 30 ml/min was controlled and measured with mass flow controllers (F-201CV-500, Bronkhorst). The O_2 flow was humidified by passing through two custom-made bubble columns. During the measurement, the pressure in the gas compartment of the GDE was measured directly in the cell by a Deltabar S pressure meter (Endress+Hauser). Before every measurement, the pressure in the catholyte compartment was increased stepwise until no more gas break-through at the GDE was observed, controlled by the backpressure of the gas outlet set by a check valve (SS-CHS2-5, Swagelok). The final pressure in both the gas and electrolyte compartments therefore resulted in approximately 450-500 mbar.

1.5 GDE materials

Specifications of the GDEs used in this study are listed in **Table S1**. The GDE materials were used as received in experiments with Cu(tmpa) as homogeneous catalyst. In the experiments where Cu(tmpa) was heterogenized on the GDE surface, the GDEs were prepared as described below.

Table S1 Overview of the different types of GDEs used, including their specifications considered to be relevant for this work.

GDE Name	Description in text	Material	MPL	Hydrophobic treatment	Thickness	Manufacturer
H23C8	Paper + MPL (hydrophobic)	Carbon Fiber Paper	Yes	Yes	230 µm	Freudenberg
H23I2	Paper (hydrophobic)	Carbon Fiber Paper	No	Yes	222 µm	Freudenberg
H23	Paper	Carbon Fiber Paper	No	No	222 µm	Freudenberg
W1S1011	Cloth + MPL	Woven Carbon Cloth	Yes	Yes	410 µm	Fuel Cell Store

1.6 GDE with drop casted ink

In the small GDE setup, an ink of Cu(tmpa) and carbon black was prepared and dropcasted onto the H23C8 Freudenberg GDE. The ink was based on a previously reported ink of Cu(tmpa)[4] and consisted of 52 wt% Cu(tmpa), 45 wt% Vulcan XC-72 and 3 wt% Nafion and was prepared from sonication of 10.40 mg Cu(tmpa), 9.0 mg Vulcan XC-72 and 10 µl of 5 wt% Nafion in 2.5 ml 50/50 EtOH/Milli-Q for 15 minutes. After sonication, 10 µl of the ink was deposited on the GDE by use of a mask with the exact diameter of the exposed electrode surface to obtain a catalyst loading of approximately 0.59 mg/cm^2. The ink was left to dry overnight before use.

1.7 GDE with spray-coated ink

For use in the GDE flow cell, an ink of Cu(tmpa) was spray-coated onto the GDE. In this ink the Nafion concentration was slightly increased compared to the dropcasted ink, while the ratio of Cu(tmpa) and Vulcan XC-72 were kept the same. The ink consisted of 49 wt% Cu(tmpa), 41 wt% Vulcan XC-72, and 10 wt% Nafion and was prepared from sonication of 21.2 mg Cu(tmpa), 18.0 mg Vulcan XC-72, and 97 µl of 5 wt% Nafion in 4.5 ml of a 50/50 isopropanol/water mixture for 30 minutes. Note that an excess of ink was prepared to account for losses during the spray-coating process.

Next, the ink was evenly sprayed onto a H23C8 Freudenberg GDE sample of 9 cm^2 that was fixed to a heating plate using a Fengda FE-134K(BD-134K) airbrush set mounted on a custom-build motorized stage. A detailed description of this setup can be found elsewhere.[3] The loading of catalyst on the GDE was determined by weighing of the GDE sample before and after the spray coating process, and resulted in a loading of 0.66 mg/cm^2.

1.8 Specifications of electrochemistry setups

In this work three different electrochemical setups have been used: a rotating disk electrode (RDE) setup, a small gas diffusion electrode (GDE) setup, and a GDE flow cell. The specifications of these setups can be found below in **Table S2**.

Table S2 Overview of the specifications of the three setups used in this work for electrochemical measurements. RDE = Rotating disk electrode, GDE = Gas diffusion electrode

Setup	WE	WE size (cm^2)	Electrolyte	Electrolyte volume (ml)	CE
RDE	Glassy Carbon (GC)	0.196	1.0 M Acetate buffer pH 4.8	40	Au (separated)
Small GDE	Carbon Paper/ Cloth (various)	0.071	3.0 M Acetate buffer pH 4.8	15	Au (separated)
GDE flow cell	Carbon Paper (Freudenberg H23C8)	3.8	1.0 M Acetate buffer pH 4.8	160 (80 ml catholyte + 80 ml anolyte)	IrO_2 on Ti, separated by a membrane

1.9 Determination of the H_2O_2 concentration

The concentration of hydrogen peroxide in the electrolyte was determined by use of reflectometric determination with the Merck Reflectoquant system in combination with 0.2-20 mg/l H_2O_2 test strips. When the H_2O_2 concentration in the electrochemical cell would exceed 20 mg/l, the samples were diluted. During electrochemical experiments, the H_2O_2 concentration was determined by taking 1.5 ml aliquots from the catalyst solution. The H_2O_2 concentration in every sample was at least measured in duplo and the error of the measurement was calculated and indicated by error bars in all graphs.

To assure the accuracy of the H_2O_2 strips in 1 M acetate buffer, the H_2O_2 concentration of multiple stock solutions was measured in presence of this buffer. To do so, a 30 wt% H_2O_2 stock solution (Sigma-Aldrich) was diluted to prepare solutions of 0.098 mM (3.33 mg/l), 0.20 mM (6.66 mg/l), and 0.39 mM (13.32 mg/l) in 1 M acetate buffer. Thereafter, the H_2O_2 concentration in these solutions was measured three times using 0.2-20 mg/l H_2O_2 test strips (**Table S3**). The concentration of hydrogen peroxide can be determined with a high

accuracy, as indicated by the trivial standard deviation. However, the results also indicate that the concentration of H_2O_2 is slightly underestimated in presence of 1 M acetate buffer, but the error is less than 5%.

Table S3 H_2O_2 concentrations measured with the Merck Reflectoquant system in combination with 0.2-20 mg/l H_2O_2 test strips in three prepared solutions of 1 M acetate buffer pH 4.8 of known H_2O_2 concentration.

Solution prepared (mg/l)	Measured #1 (mg/l)	Measured #2 (mg/l)	Measured #3 (mg/l)	Average (mg/ml)	Standard deviation (mg/l)	Error (%)
3.3	3.2	3.1	3.1	3.2	0.047	4.4
6.7	6.5	6.2	6.0	6.3	0.21	4.8
16.7	15.9	17.0	16.1	16.4	0.48	1.6

2. *iR* Compensation

The resistance, i.e. *iR* drop, of the small GDE cell was determined to compensate for the R_u (uncompensated resistance) of the electrolyte solution.[5] The resistance could be obtained from the high-frequency part of electrochemical impedance measurements. As can be seen in **Figure S1a**, the R_u of the setup is greatly

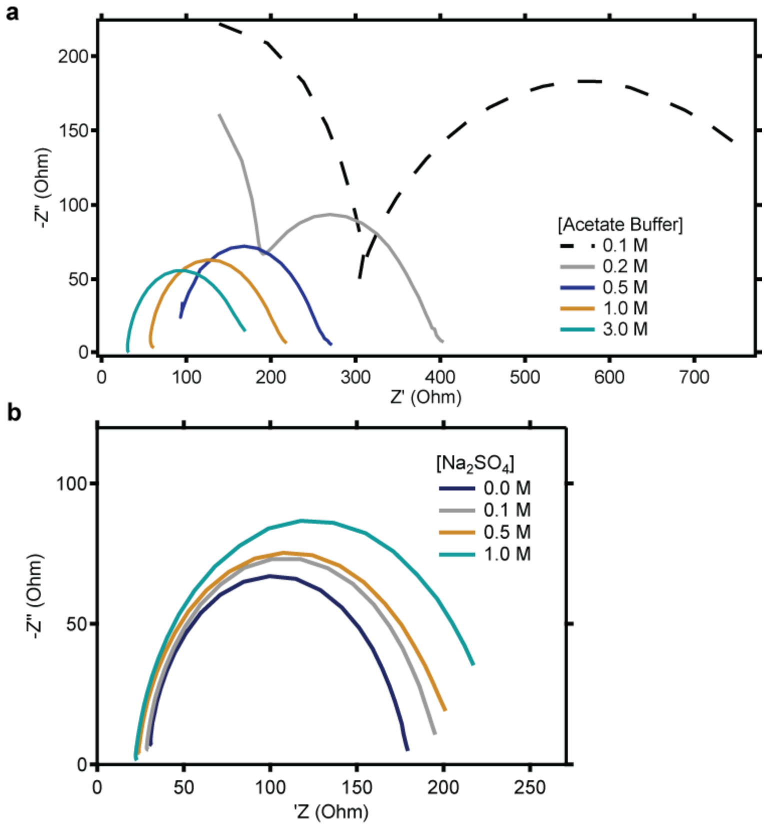

Figure S1 Nyquist plots measured in the small GDE cell to determine the uncompensated resistance of the setup using a frequency range from 100 – 1000000 Hz. **a**) Nyquist plot for varying concentrations of acetate buffer between 0.1 M and 3.0 M and **b**) Nyquist plot measured in presence of 3.0 M acetate buffer and additional supporting electrolyte Na_2SO_4 with a concentration between 0.0 to 1.0 M. Conditions: 0.3 mM Cu(tmpa), 0.1 V vs. RHE, pH 4.8, O_2 flow field, H23C8 GDE.

decreased by increasing the buffer concentration. A similar effect is obtained when Na_2SO_4 is added as a supporting electrolyte, although the effect is substantially smaller (see **Figure S1b**). Following from these measurements a high buffer concentration of 3.0 M was used in the small GDE setup to maintain good conductivity and reduce the uncompensated resistance.

The R_u does not depend on the applied potential, hence *iR* compensation could be applied after all electrochemical measurements (see **Figure S2a**). It should be noted that when a dynamic potential is involved, like in CVs, part of the current may be non-faradaic at high scan rates, while *iR* compensation should only be applied to the faradaic current to prevent overcompensation. However, in our case the same CVs were obtained independent of whether *iR* compensation was done during measurements or *iR* correction after measurements, which indicates that our method compensates for the complete faradaic current (see **Figure S2b**).

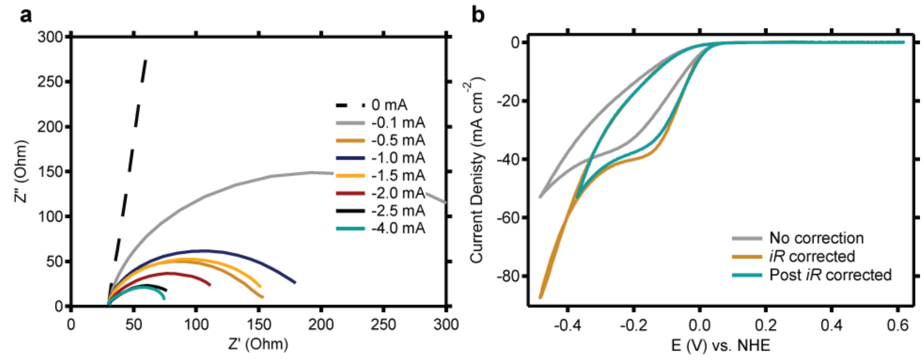

Figure S2 a) Nyquist plot measured in the small GDE cell at various currents between 0 mA and -4.0 mA,, using a frequency range from 100 – 1000000 Hz, from which it is evident that the uncompensated resistance does not change with the applied potential. **b**) CV measurements in presence of Cu(tmpa) with no *iR* correction (grey) and with *iR* correction (29 Ω) applied before the CV measurement (orange) and after the CV measurement (light blue). Conditions: 0.3 mM Cu(tmpa), 0.1 V vs. RHE, 3.0 M acetate buffer pH 4.8, O_2 flow field, H23C8 GDE.

3. CV Measurements in the Small GDE Cell

Three consecutive CV scans of the ORR activity of Cu(tmpa) in the small GDE cell were recorded. In the measurements of **Figure S3a** the electrolyte solution was not stirred, whereas this was the case in **Figure S3b**. The decrease in current in **Figure S3a** indicates that a species gets depleted during catalysis, which can be prevented by stirring of the electrolyte solution.

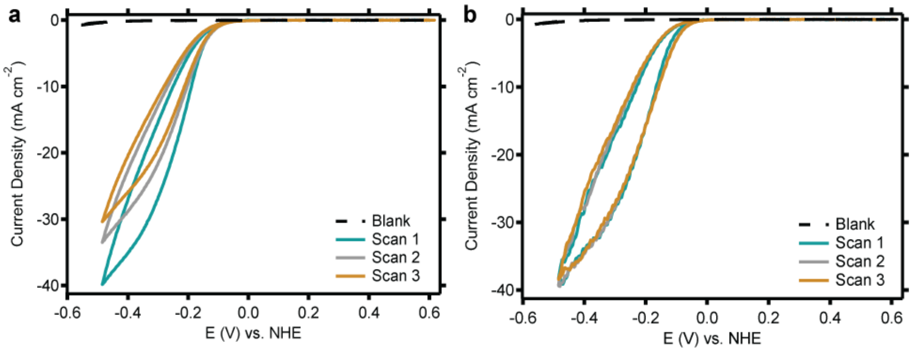

Figure S3 CV measurements recorded in the small GDE cell to investigate catalysis during 3 consecutive scans **a**) without and **b**) with stirring of the electrolyte solution. Conditions: 0.3 mM Cu(tmpa), 3.0 M acetate buffer pH 4.8, O_2 flow field, H23C8 GDE, 100 mV/s scan rate, full iR compensation.

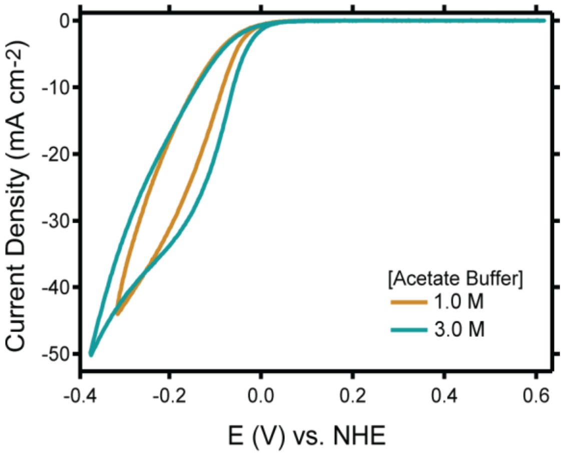

Figure S4 CV measurements recorded in the small GDE in presence of 1.0 M (orange) or 3.0 M (blue) of buffer. Conditions: 0.3 mM Cu(tmpa), 1.0 or 3.0 M acetate buffer pH 4.8, O_2 flow field, H23C8 GDE, 100 mV/s scan rate, full *iR* compensation

3.1 Effect of pH

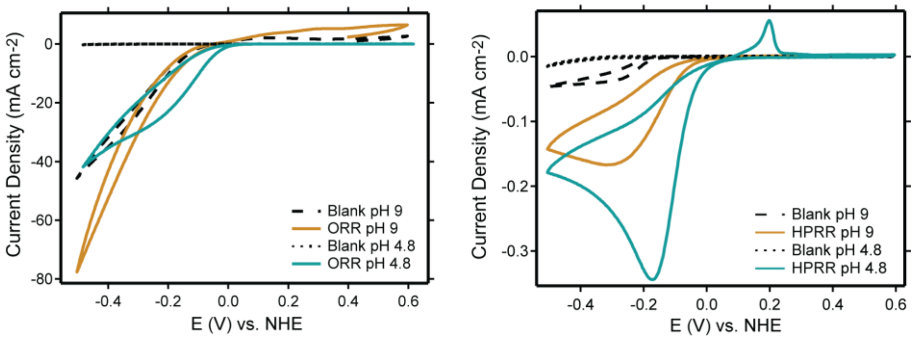

Figure S5 CV measurements recorded in the small GDE cell to investigate catalysis of the ORR and HPRR at different pH. **a**) Measurements in presence of an O_2 flow field at pH 9 (orange) or pH 4.8 (blue) and **b**) measurements in presence of an Ar flow field, deoxygenated electrolyte and H_2O_2 at pH 9 (orange) or pH 4.8 (blue). Conditions: 0.3 mM Cu(tmpa), 3.0 M acetate buffer pH 4.8 or 3.0 M sodium acetate pH 9, O_2 flow field or Ar flow field + 1.1 mM H_2O_2, H23C8 GDE, 100 mV/s scan rate, full *iR* compensation.

4. GDE Flow Cell

After assembly of the GDE flow cell, LSV and CV measurements were recorded to confirm the proper assemblance of the cell and record blank measurements of the GDE surface. Blank measurements in absence of catalyst were recorded until the measured current stabilized. Typically, this involved recording 5 LSV scans between 0.6 V and -0.8 V vs. NHE. Thereafter, 0.3 mM Cu(tmpa) was dissolved in the catholyte solution and the same procedure was repeated until the catalytic current stabilized and the chronopotentiometry measurements would be started.

4.1 4-hour electrolysis

A 4-hour electrolysis experiment was carried out in the GDE flow cell at a current density of -10.3 mA/cm^2 to generate H_2O_2 (**Figure S6**). Over the course of this measurement both the FE and applied potential were maintained. However, CV measurements of the GDE after electrolysis showed clear changes compared to CVs before the measurement (**Figure S6c**). We hypothesize that the broadening of the catalytic trace can be assigned to the increase of the accessible electrode surface area, as discussed in section **7.1** below. In addition,

two new oxidation events are observed between 0.2 – 0.6 V vs. NHE. The stability of Cu(tmpa) was further investigated in section **7.2**, indicating that the concentration of Cu(tmpa) was decreased during the measurement. Hence, we can assign these new oxidation events to the oxidation of metallic copper that gets deposited onto the electrode during catalysis.

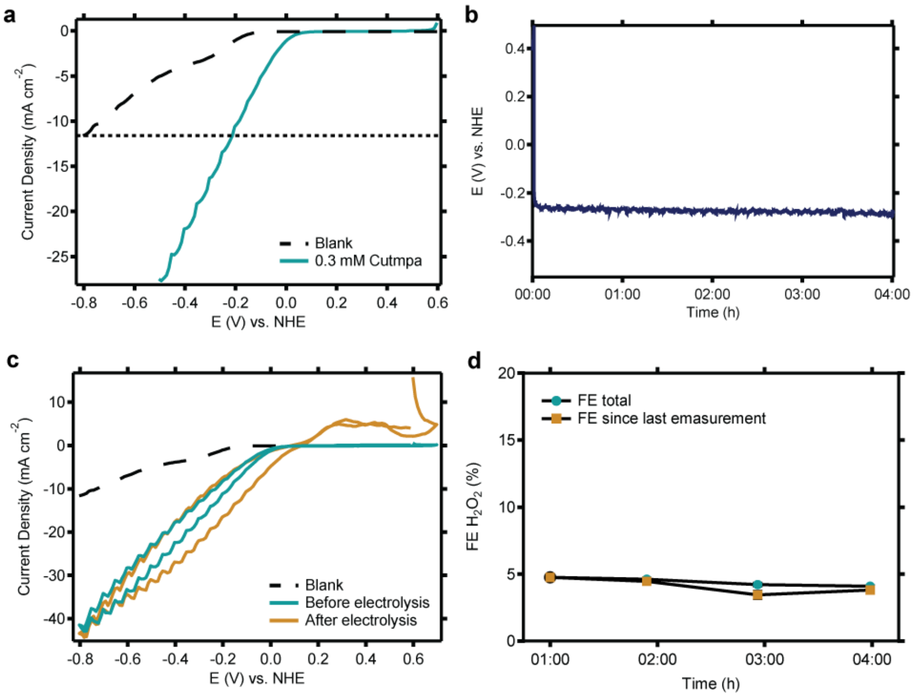

Figure S6 Overview of experiments involved with the 4-hour electrolysis in the GDE flow cell. **a**) LSV measurements recorded in absence (black dashed line) and presence of catalyst (light blue) and the current density selected for electrolysis indicated by a horizontal dotted line. **b**) voltammogram of the measured potential from the electrolysis at -10.3 mA/cm^2. **c**) CV measurements of the GDE in presence of catalyst before and after 4-hour electrolysis at -10.3 mA/cm^2. **d**) Faradaic efficiency (FE) towards H_2O_2 during the 4-hour electrolysis determined as the total FE and the FE measured since the last data point. Conditions: 0.3 mM Cu(tmpa), 1.0 M acetate buffer pH 4.8, O_2 flow field, H23C8 GDE, 100 mV/s scan rate, no *iR* compensation, O_2 flow 30 ml/min, liquid flow 30 RPM. The noise in the voltammograms can be attributed to the the electrolyte pump.

4.2 Effect of liquid and gas flow rates

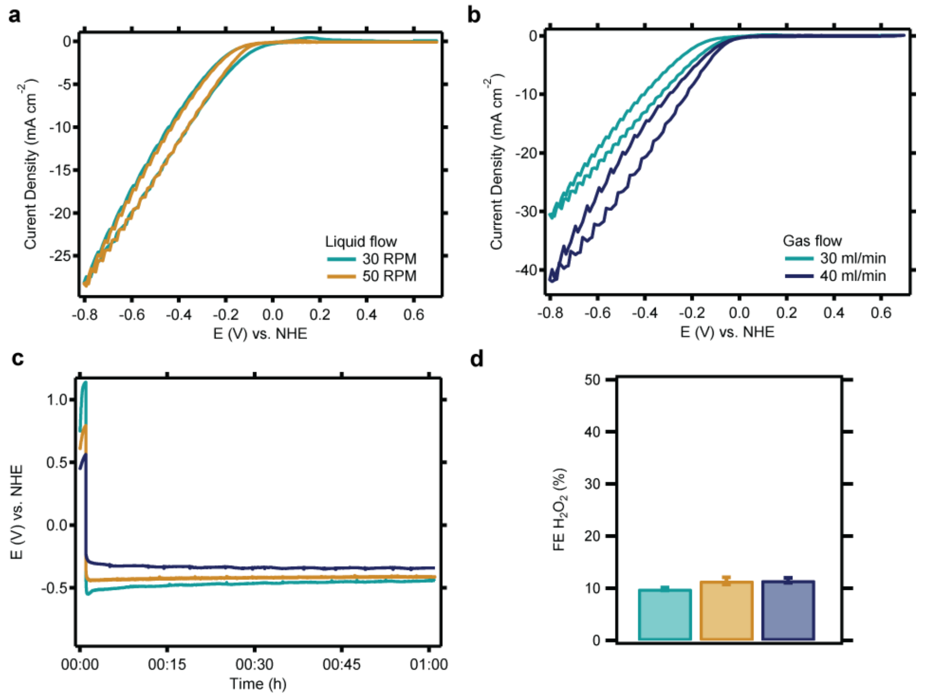

Figure S7 Overview of experiments involved with electrolysis in the GDE flow cell. **a**) CV measurements recorded with a different liquid flow rate of 30 RPM (light blue) and 50 RPM (orange) at a gas flow rate of 30 ml/min. **b**) CV measurements recorded with a different gas flow rate of 30 ml/min (light blue) and 40 ml/min (dark blue) at a liquid flow rate of 30 RPM. **c**) voltammogram of the measured potential during electrolysis at -10.3 mA/cm^2 for all three conditions. **d**) Overall Faradaic efficiency (FE) towards H_2O_2 of this electrolysis under all three measurements. Conditions: 0.3 mM Cu(tmpa), 1.0 M acetate buffer pH 4.8, O_2 flow field, H23C8 GDE, 100 mV/s scan rate, no iR compensation. In all graphs light blue trace corresponds to a liquid flow rate of 30 RPM and gas flow rate of 30 ml/min, orange trace corresponds to liquid flow rate of 50 RPM and gas flow rate of 30 ml/min, and dark blue trace corresponds to liquid flow rate of 30 RPM and gas flow rate of 40 ml/min. The noise in the voltammograms can be attributed to the electrolyte pump.

.

4.3 Effect of current density

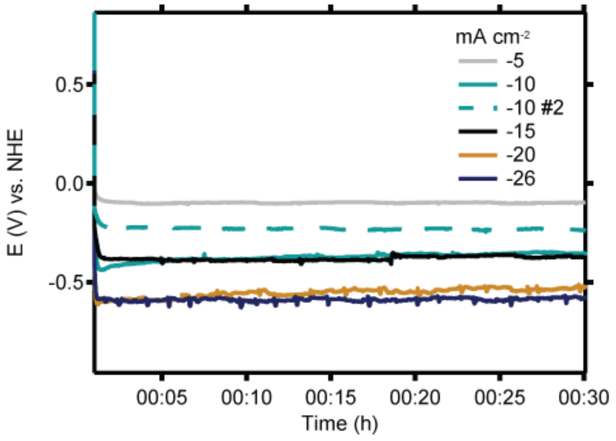

Figure S8 Crude data belonging to Figure 4 in the main text. Voltammogram of the measured potential at various current densities that have been recorded in a random order (see Figure 4) in the flow electrolysis GDE setup. The current density of -10 mA/cm^2 was measured two times, once at the beginning of the sequence, and once at the very end when the H_2O_2 concentration within the system has built up. Conditions: 0.3 mM Cu(tmpa), 1.0 M acetate buffer pH 4.8, O_2 flow field, H23C8 GDE, no iR compensation, O_2 flow 30 ml/min, liquid flow 30 RPM.

To obtain better insight into the reproducibility of the experiments carried out in the GDE flow setup, electrolysis at -10 mA/cm^2 was repeated, and the results of are shown in the voltammogram in Figure S9. These results indicate there is a slight deviation in the required voltage between two similar experiments of approximately 110 mV. The FE towards H_2O_2 corresponding to these experiments was 8.6% for Exp. 1 and 9.8% for Exp. 2. Consequently, we can estimate the standard deviation in the measured voltage to be approximately ±78 mV and in the FE to H_2O_2 to be approximately ±0.8%. Both these deviations are relatively low, and we have assumed that the error margin for all other experiments would be similarly small.

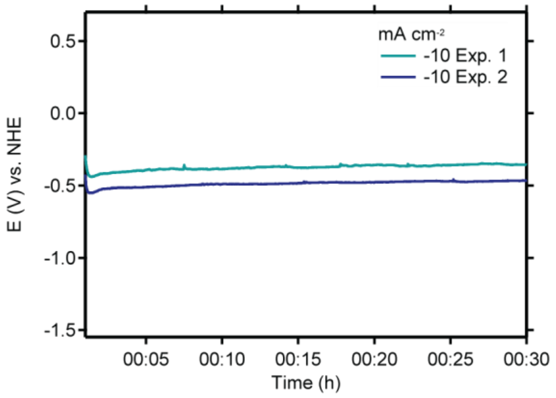

Figure S9 Voltammogram of two electrolysis experiments at a current density of -10 mA/cm^2 measured on two different days. Conditions: 0.3 mM Cu(tmpa), 1.0 M acetate buffer pH 4.8, O_2 flow field, H23C8 GDE, no iR compensation, O_2 flow 30 ml/min, liquid flow 30 RPM.

5. Measurements with Heterogenized Catalyst

In **Figure 4** (main text) CV measurements of Cu(tmpa) heterogenized onto the GDE surface by drop casting are shown. Details of the catalyst ink and GDE preparation can be found in section **1.2**. The redox couples measured in **Figure 4b** were first recorded in presence of Ar. Thereafter, the flow field was changed to an O_2 atmosphere, and 10 catalytic ORR CVs were recorded, of which the first scans can be seen in **Figure 4c**. After catalysis the flow field was switched back to Ar and the redox events were measured again. The resulting data in **Figure 4d** indicates that after catalysis the redox couple is diminished, and a new oxidation event appears around 0.3 V vs. NHE. The combination of both these changes suggests that part of the catalyst is broken down during catalysis, and most likely metallic copper deposits is generated that contributes to these oxidation events.

6. Cu(tmpa) Redox Couple

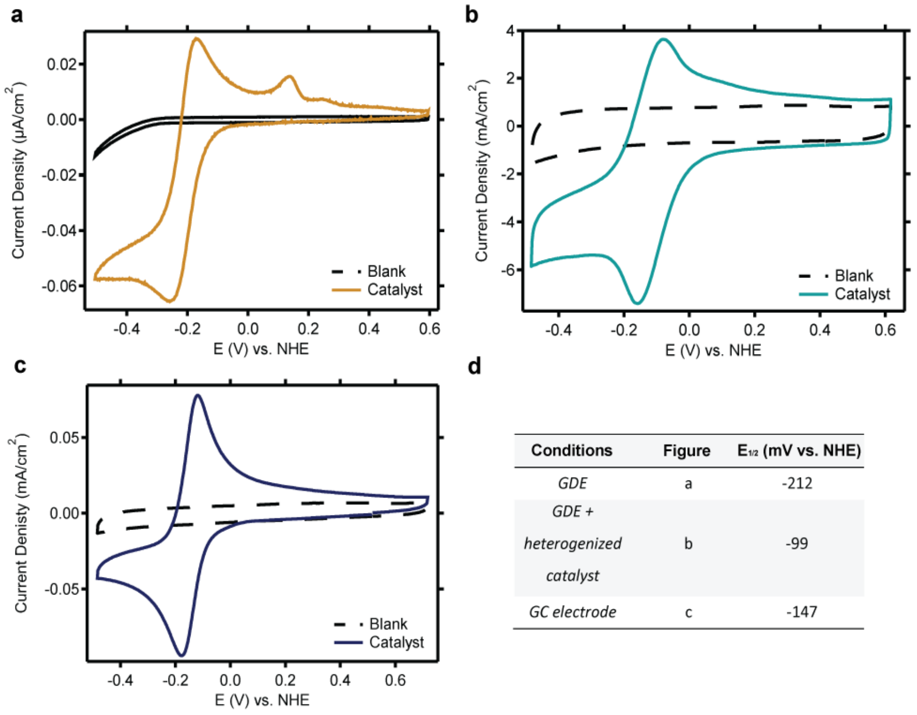

Conditions	Figure	$E_{1/2}$ (mV vs. NHE)
GDE	a	-212
GDE + heterogenized catalyst	b	-99
GC electrode	c	-147

Figure S10 CV measurements recorded of the Cu(tmpa) Cu^{II}/Cu^{I} redox couple in different conditions. **a**) In a GDE setup with 0.3 mM Cu(tmpa) in solution, **b**) in a GDE setup with 10 µl of ink drop casted onto the GDE, and **c**) using a glassy carbon working electrode with 0.3 mM Cu(tmpa). In **d**) the different $E_{1/2}$ values are given. Conditions: 0.3 mM Cu(tmpa) or 10 µl catalyst ink with 0.59 mg/cm² catalyst loading. Acetate buffer of pH 4.8 with a concentration of 3.0 M (figure a + b) or 0.1 M (figure c), Ar atmosphere or Ar flow field, H23C8 GDE (figure a + b) or GC electrode (figure c), 100 mV/s scan rate, no iR compensation.

7. Stability Investigations

7.1 Stability of the GDE

The stability of the GDE in absence of catalyst (blank) was investigated in the GDE cell (**Figure S10a-b**). Interestingly, the catalytic current drastically increases when the lower limit of the CV is set to -0.7 V vs. NHE, as opposed to a lower limit of only -0.5 V vs. NHE. Most likely the increase in current is caused by an increase

of the electrochemical surface area. This result can be linked to the observation that on carbon-based GDEs negative potentials drive a potential-induced flooding, changing the wettability of the GDE, and therefore the electrochemical surface area.[6] To confirm this, the electrodes from **Figure S11a-b** were used to record the catalytic ORR activity of Cu(tmpa). The CV measurements shows that the catalytic current generated by Cu(tmpa) is much larger on the electrode that was scanned to -0.7 V vs. NHE, confirming the increase in the electrochemical surface area (**Figure S11c**).

Additionally, more experiments were conducted in presence of catalyst (**Figure S10c**). When the lower limit of the CV measurement is set to -0.5 V vs. NHE, the catalytic current decreases as expected. When the limit of the CV is however set to a more negative potential of -0.7 V vs. NHE, the measured current largely increases and a large oxidation event arises above 0.0 V vs. NHE. The origin of this sharp catalytic current and new oxidation event could not be determined, but remind of the oxidation peaks that arise in CV measurements after electrolysis (see **Figure S6c**). It should be noted that no *iR* compensation was applied to the measurements of **Figure S11**, as it is uncertain whether the surface and resistance of the GDE remain the same throughout the measurements.

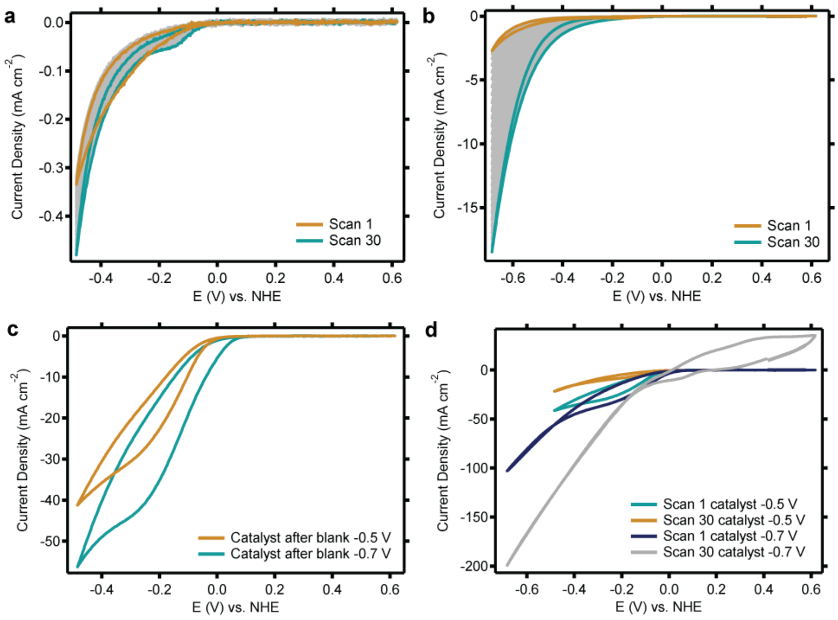

Figure S91 CV measurements of a blank GDE during 30 consecutive scans with the lower limit of the CV set to **a**) -0.5 V vs. NHE or **b**) -0.7 V vs. NHE. **c**) CV measurements of Cu(tmpa) in solution using the GDEs from figure a and b on which 30 CV scans were recorded with a lower limit of -0.5 V (orange) or -0.7 V (light blue) vs. NHE. **d**) CV measurements of Cu(tmpa) during 30 consecutive scans with the lower limit of the CV set to -0.5 V vs. NHE (scan 1 light blue vs scan 30 orange) or set to -0.7 V vs. NHE (scan 1 dark blue vs scan 30 grey). Conditions: 0.3 mM Cu(tmpa), 3.0 M acetate buffer pH 4.8, O_2 flow field, H23C8 GDE, 100 mV/s scan rate, no iR compensation was done as the system changes throughout the experiment.

7.2 Catalyst stability in CV measurements

The stability of the homogeneous Cu(tmpa) catalyst during catalysis was investigated by recording CV measurements in the small GDE cell. No substantial change is observed when CV measurements were recorded periodically over the course of two hours (**Figure S12a**). Therefore, we can conclude that Cu(tmpa) remains active, and the electrochemical surface area of the GDE does not undergo any time-induces changes in absence of an applied potential.

In **Figure S12b**, 25 consecutive CV scans of Cu(tmpa) were recorded while the solution was stirred, as to avoid depletion of substrates. This clearly shows that the catalytic current increases over the course of the experiment. A reasoning for this is provided either by an increase of the electrochemical surface area of the GDE, or by the formation of a catalytically active deposit on the GDE surface due to breakdown of the catalyst during catalysis. To investigate the latter, the electrolyte was removed from the GDE cell after these 25 scans, and the surface of the GDE was cleaned with Milli-Q. Thereafter, the ORR activity of the rinsed electrode was measured, from which it is evident that the GDE is only slightly more active than the blank GDE at the start of the measurement. Therefore, the formation of catalytically active species does not explain the increased catalytic, and likely the increased electrochemical surface area of the GDE is the cause. This observation is in line with the measurement in section **7.1** that show that the GDE surface is prone to change during CV measurements.

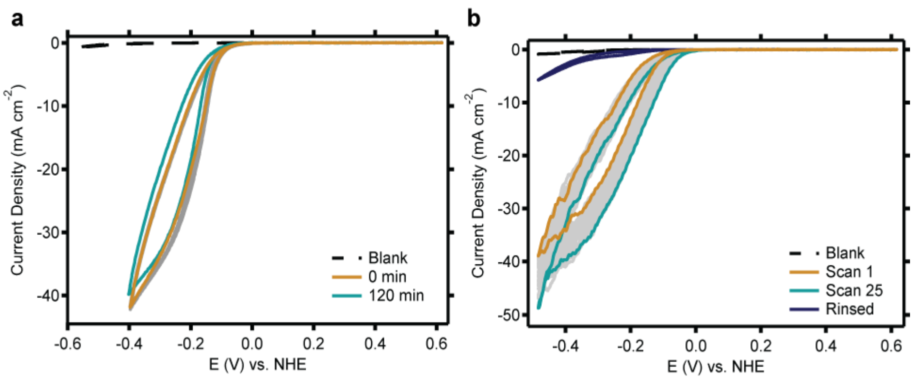

Figure S102 CV measurements of homogeneous Cu(tmpa) in the small GDE cell to investigate the stability of the catalyst during the ORR. **a**) CV measurements recorded at the start of the day (orange), every 30 minutes (grey) and after two hours (light blue), showing no significant differences. And **b**) 25 consecutive CV scans recorded in presence of catalyst (scan 1 orange, scan 25 light blue), while the solution was stirred, compared to measurements of the same GDE in absence of catalyst, before (black dashed line) and after catalysis (dark blue). Conditions: 0.3 mM Cu(tmpa), 3.0 M acetate buffer pH 4.8, O_2 flow field, H23C8 GDE, 100 mV/s scan rate, full iR compensation in figure a, no iR compensation in figure b

7.3 Catalyst stability in electrolysis

SEM images of a GDE were recorded before and after its use in electrolysis in the flow cell (**Figure S14**), corresponding to the measurements in **Figure S6**. This analysis shows there are no visible differences between the samples. The elemental mapping of Cu obtained by EDX does not indicate the formation of any metallic copper on the GDE during catalysis, which contrast the observation in **Figure S6**. This discrepancy can be

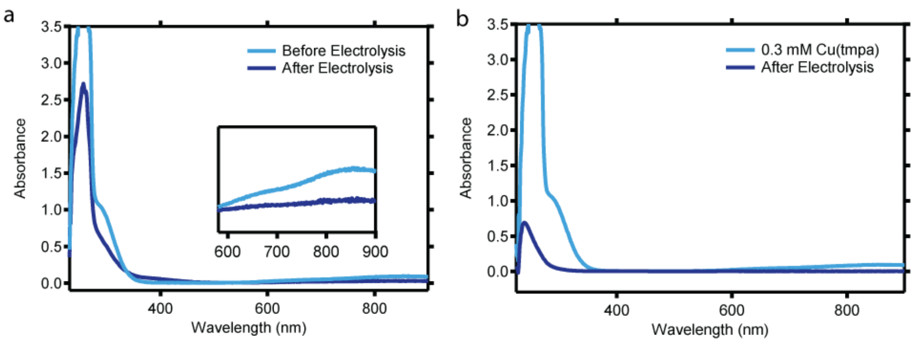

Figure S11 UV-vis spectra recorded of the electrolyte used in the GDE flow cell **a**) before and after 4 hours of electrolysis at an applied current density -10.3 mA/cm^2 with 0.3 mM Cu(tmpa) as a homogeneous catalyst in solution, and **b**) after electrolysis of 4 hours at -10-20 mA/cm^2 using a GDE with spray-coated catalyst, compared to a UV-vis spectrum of 0.3 mM Cu(tmpa) in solution. Conditions: 1.0 M acetate buffer pH 4.8.

deduced to the fact that after electrolysis CV scans were recorded, hence the deposited metallic copper was likely oxidized to Cu(II) and stripped from the surface.

Next, SEM measurements of the Cu(tmpa) spray-coated GDE were recorded before and after use in electrolysis in the flow cell (**Figure S15**). The GDE was used for the electrolysis measurements depicted in **Figure S4**. A clear difference can be seen between the sample before and after electrolysis, as the catalyst ink cracked after electrolysis. The elemental mapping of Cu, obtained by EDX, does not indicate any change in the concentration of copper on the GDE surface before and after catalysis, which indicates that the copper has remained confined in the ink. This observation is in line with the UV-vis measurement in **Figure S13b**, which indicates that no Cu(tmpa) has leached out of the ink into the electrolyte solution.

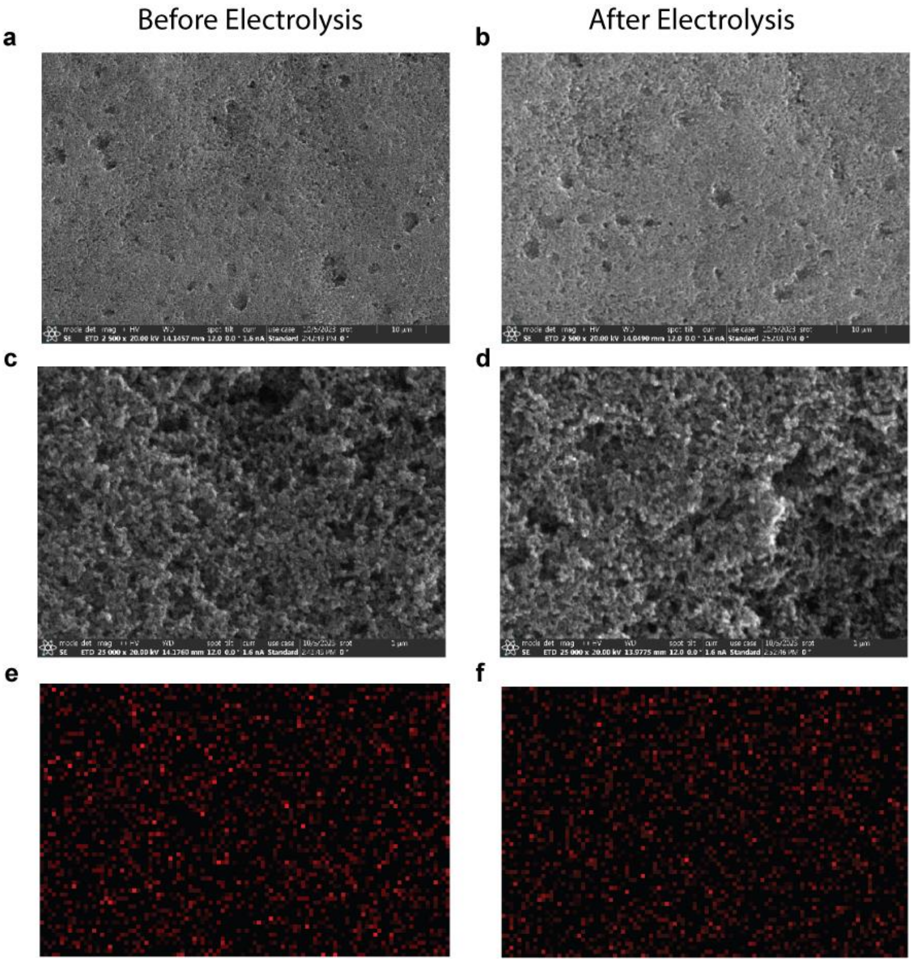

Figure S12 SEM measurements of the GDE before (a-c-e) and after (b-d-f) electrolysis measurements. **a – b**) image zoom 2500 x, **c-d**) image zoom 25000 x, **e-f**) elemental mapping of net Cu counts.

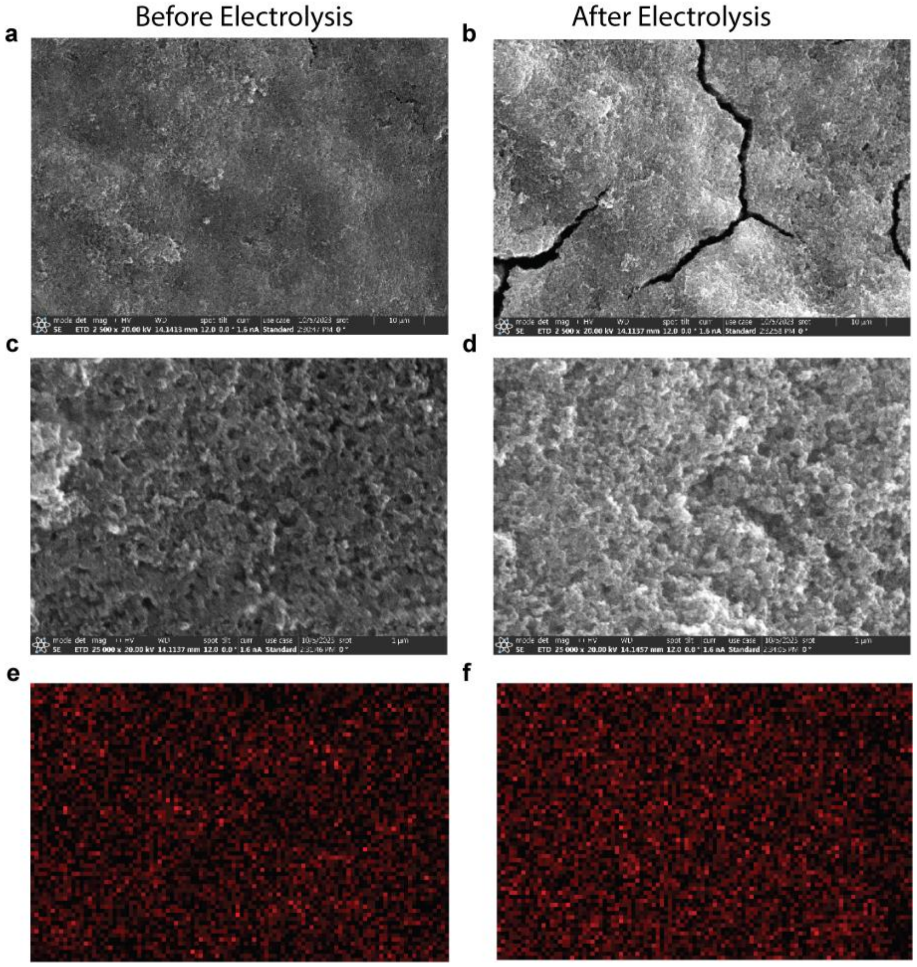

Figure S13 SEM measurements of a GDL with spray coated Cu(tmpa) sample before (**a**-**c**-**e**) and after (**b**-**d**-**f**) electrolysis measurements. **a** – **b**) image zoom 2500 x, **c**-**d**) image zoom 25000 x, **e**-**f**) elemental mapping of net Cu counts.

7.4 Stability of H_2O_2 solutions

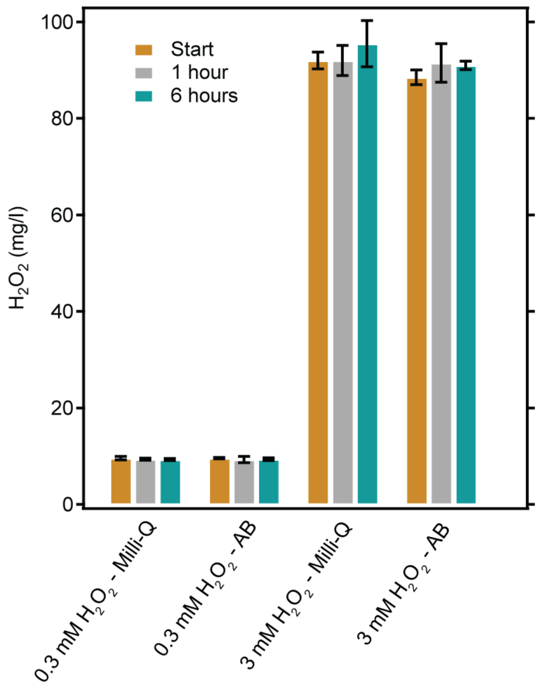

Figure S16 The concentration of H_2O_2 determined in solutions of 0.3 mM or 3 mM H_2O_2 in Milli-Q or AB, measured at the start (yellow), after 1 hour (grey), and after 6 hours (blue). AB = Acetate Buffer. Conditions: 0.1 M acetate buffer pH 4.8. Error bars based on three measurements of the same solution

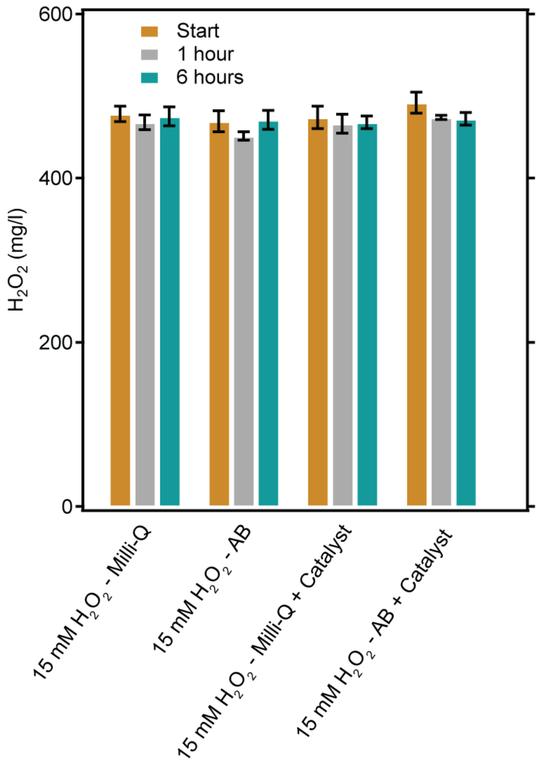

Figure S14 The concentration of H_2O_2 determined in solutions of 0.3 mM or 3 mM H_2O_2 in Milli-Q or AB, measured at the start (yellow), after 1 hour (grey), and after 6 hours (blue). AB = Acetate buffer. Conditions: 0.1 M acetate buffer pH 4.8, 0.3 mM Cu(tmpa). Error bars based on three measurements of the same solution.

8. H_2O_2 Generation in Different Setups

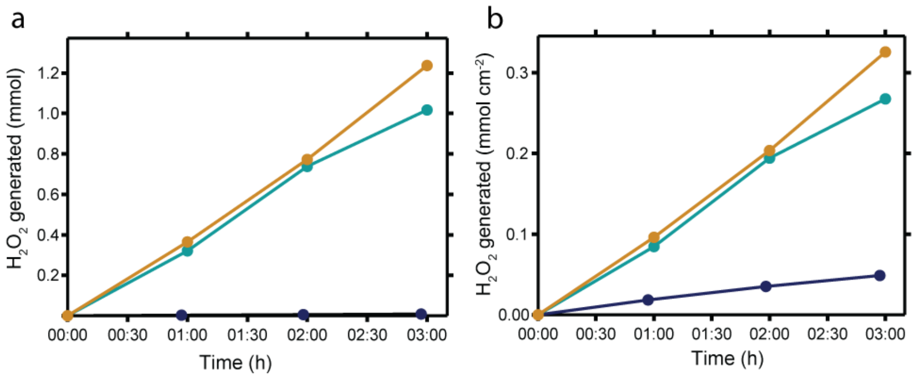

Figure S158 H_2O_2 generated during electrolysis in **a**) mmol or **b**) mmol/cm^2 in different setups, RDE (dark blue), GDE + homogeneous catalyst (light blue), GDE spray coated catalyst (orange). Conditions: RDE: 0.1 M acetate buffer pH 4.8, 0.005 mM Cu(tmpa), 1600 RPM, O_2 atmosphere, GC electrode. GDE homogeneous catalyst: 1.0 M Acetate buffer pH 4.8, H23C8 GDE, -20 mA/cm^2, 0.3 mM Cu(tmpa). GDE heterogeneous catalyst (orange): 1.0 M acetate buffer pH 4.8, H23C8 GDE, -20 mA/cm^2, 0.66 mg/cm^2 Cu(tmpa). Data for the RDE measurements obtained from reference 2.

9. References

(1) Langerman, M.; Hetterscheid, D. G. H. Fast Oxygen Reduction Catalyzed by a Copper(II) Tris(2-pyridylmethyl)amine Complex through a Stepwise Mechanism. *Angew. Chem. Int. Ed.* **2019**, *58* (37), 12974-12978. DOI: 10.1002/anie.201904075.

(2) van Langevelde, P. H.; Hetterscheid, D. G. H. Selective electrochemical H_2O_2 production by a molecular copper catalyst: A crucial relation between reaction rate and mass transport. *Chem Catal.* **2024**, *4* (8) 101069. DOI: 10.1016/j.checat.2024.101069.

(3) Baumgartner, L. M.; Koopman, C. I.; Forner-Cuenca, A.; Vermaas, D. A. When Flooding Is Not Catastrophic-Woven Gas Diffusion Electrodes Enable Stable CO_2 Electrolysis. *ACS Appl. Energy Mater.* **2022**, *5* (12), 15125-15135. DOI: 10.1021/acsaem.2c02783.

(4) Thorseth, M. A.; Letko, C. S.; Tse, E. C.; Rauchfuss, T. B.; Gewirth, A. A. Ligand effects on the overpotential for dioxygen reduction by tris(2-pyridylmethyl)amine derivatives. *Inorg. Chem.* **2013**, *52* (2), 628-634. DOI: 10.1021/ic301656x.

(5) Zheng, W. iR Compensation for Electrocatalysis Studies: Considerations and Recommendations. *ACS Energy Lett.* **2023**, *8* (4), 1952-1958. DOI: 10.1021/acsenergylett.3c00366.

(6) Yang, K.; Kas, R.; Smith, W. A.; Burdyny, T. Role of the Carbon-Based Gas Diffusion Layer on Flooding in a Gas Diffusion Electrode Cell for Electrochemical CO_2 Reduction. *ACS Energy Lett.* **2020**, *6* (1), 33-40. DOI: 10.1021/acsenergylett.0c02184.
